# *De novo* assembly and next-generation sequencing to analyse full-length gene variants from codon-barcoded libraries

**DOI:** 10.1038/ncomms9351

**Published:** 2015-09-21

**Authors:** Namjin Cho, Byungjin Hwang, Jung-ki Yoon, Sangun Park, Joongoo Lee, Han Na Seo, Jeewon Lee, Sunghoon Huh, Jinsoo Chung, Duhee Bang

**Affiliations:** 1 Department of Chemistry, Yonsei University, Seoul 120-749, Republic of Korea; 2 College of Medicine, Seoul National University, Seoul 110-744, Republic of Korea; 3 Department of Chemistry, Korea Military Academy, Seoul 139-799, Republic of Korea; 4 Department of Chemistry, University of Oxford, Oxford OX1 3TA, UK; 5 Department of Chemical and Biomolecular Engineering, University of California, Berkeley, California 94720-1462, USA

## Abstract

Interpreting epistatic interactions is crucial for understanding evolutionary dynamics of complex genetic systems and unveiling structure and function of genetic pathways. Although high resolution mapping of *en masse* variant libraries renders molecular biologists to address genotype-phenotype relationships, long-read sequencing technology remains indispensable to assess functional relationship between mutations that lie far apart. Here, we introduce JigsawSeq for multiplexed sequence identification of pooled gene variant libraries by combining a codon-based molecular barcoding strategy and *de novo* assembly of short-read data. We first validate JigsawSeq on small sub-pools and observed high precision and recall at various experimental settings. With extensive simulations, we then apply JigsawSeq to large-scale gene variant libraries to show that our method can be reliably scaled using next-generation sequencing. JigsawSeq may serve as a rapid screening tool for functional genomics and offer the opportunity to explore evolutionary trajectories of protein variants.

## Abstract (web summary)

This paper described a new and efficient method for *de novo* assembly of multiple DNA sequence information from mutagenized clone libraries. Using codon-barcoded libraries and calling the method JigsawSeq, the authors overcome limitations of short-read sequencing assembly from next-generation sequencing.

## Graphical abstract

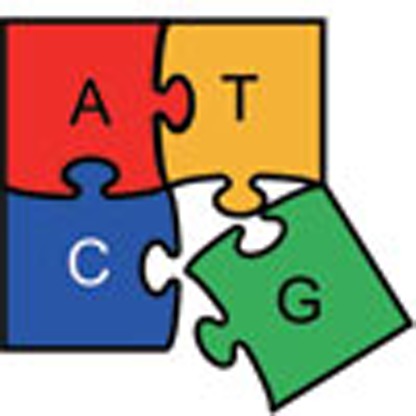

Functional analysis of highly complex mutant protein libraries is a powerful tool for deep mutational scanning of potential sequence-function relationships. Typically, random mutagenesis libraries are created by error-prone polymerase chain reaction (PCR)^1^, random shuffling^2^, programmed mutagenesis^3^,^4^ or assembly of synthetic oligonucleotides^5^. Careful scrutiny of variant libraries may enable us to delineate the crucial role for each sequence variation, leading to the identification of changes in protein activities. However, as the number of library sequences exceeds hundreds of clones, sequence verification of variants becomes limited in scalability, and only a few clones are subjected to further analysis via conventional cloning and Sanger sequencing^6^. Although the characterization of a few ‘selected' clones has shown great utility for discovering sequence information bearing top characteristic functions, the small number of clones may not represent the full-spectrum of sequence space that may contain crucial mutations that are potentially adaptive in a given environment^7^.

High-throughput sequencing offers enormous benefits in sequencing analysis of variant libraries because of its unprecedented accuracy and processing capability. Comprehensive mapping of promoter regions or protein domains using next-generation sequencing (NGS), or ‘deep mutational scanning', has previously been performed. Pioneering explorations of these large-scale analysis techniques mainly focused on small genomic regions that can be sequenced via short-read NGS platforms^8^,^9^,^10^,^11^. Recently, numerous studies have been undertaken to analyse longer genomic region on a library scale^12^,^13^. For example, the PacBio RS II platform^14^, which has been adapted for *de novo* assembly of sequencing reads for genome contiguity, was utilized to analyse libraries with longer region. However, PacBio is still expensive for general laboratory use and errors should be resolved via multi-consensus read, requiring large number of reads to profile large variant population.

The two general categories of short-read sequence assembly based on hierarchical structuring are as follows: reference-based and *de novo* approaches. The former is more straightforward as it aligns the reads and merges them into final contigs^15^,^16^. However, the aligner must tolerate imperfect mapping to avoid missing true joins. Error tolerance causes false assemblies making chimeric contigs. This leads to problems especially when the library is highly polymorphic. Barcode assignment to each reads would provide an alternative solution for tracing where original molecule came from. Promising approach utilizing both barcoding and sub-assembly has been pursued^17^,^18^. These researchers utilized several nested forward primers and common random barcode primers to generate templates for assembly. Subsequently, overlap-layout-consensus assembly was performed to resolve long-range information. However, PCR using random primers tends to create biases thereby over-representing certain templates and preventing perfect assembly^19^. As regions to be analysed increase in length, serial tiling of primers over the target is required such that individual synthesis of the nested primers would also increase.

Many *de novo* assembly algorithms^20^,^21^ have shown promise especially for analysing significantly altered genomes. An elegant de Bruijn^22^ -based graph provides an efficient solution for whole-genome assembly. The graph consists of vertices (nodes) defined by *k*-mer nucleotides and edges (suffix to prefix *k*-1 mer overlaps between reads). Unlike genome assembly which can be solved in linear time, transcriptome assembly is more complex and computationally challenging because alternative splicing produces many isoforms that requires traversing a multi-path graph^23^. Likewise, disentangling individual molecules from closely related species in a metagenome library or error-prone PCR library is confounded by genomic diversity and non-uniform coverage of the population. Variability can be introduced based on compositional bias of sequencing technologies and cellular copy number variation.

To overcome these limitations, we develop a novel *de novo* assembler called JigsawSeq. The method utilizes codon-barcoded library and cost-effective Illumina NGS platform^24^ for profiling the full-spectrum of multiple gene variant libraries via computational assembly of short reads (Fig. 1a,b). Briefly, one protein sequences can be reverse-translated to make a diverse, randomized library of synonymous gene sequences that can be constructed by incorporating degenerate nucleotides as barcodes during *in vitro* library synthesis. When the gene libraries (or gene variants) are subjected to random shearing for NGS preparation, even short NGS reads can be connected based on randomized codons as barcodes to retrace back original sequences. We demonstrate general applicability of our method by (i) analysing libraries of pooled gene variants, (ii) implementing different cloning and library construction methods and (iii) extensive simulations to show scalability by analysing thousands of gene variants. We show that our algorithm has high sensitivity and specificity over a wide range of genes and experimental procedures. Comparing real and simulated data, our results highlight the fact that JigsawSeq can be potentially applied even to evaluating larger (100,000) libraries overcoming the size limit of the region to be analysed.

## Results

### Library construction of gene variants using codon barcode

**Figure 1:**
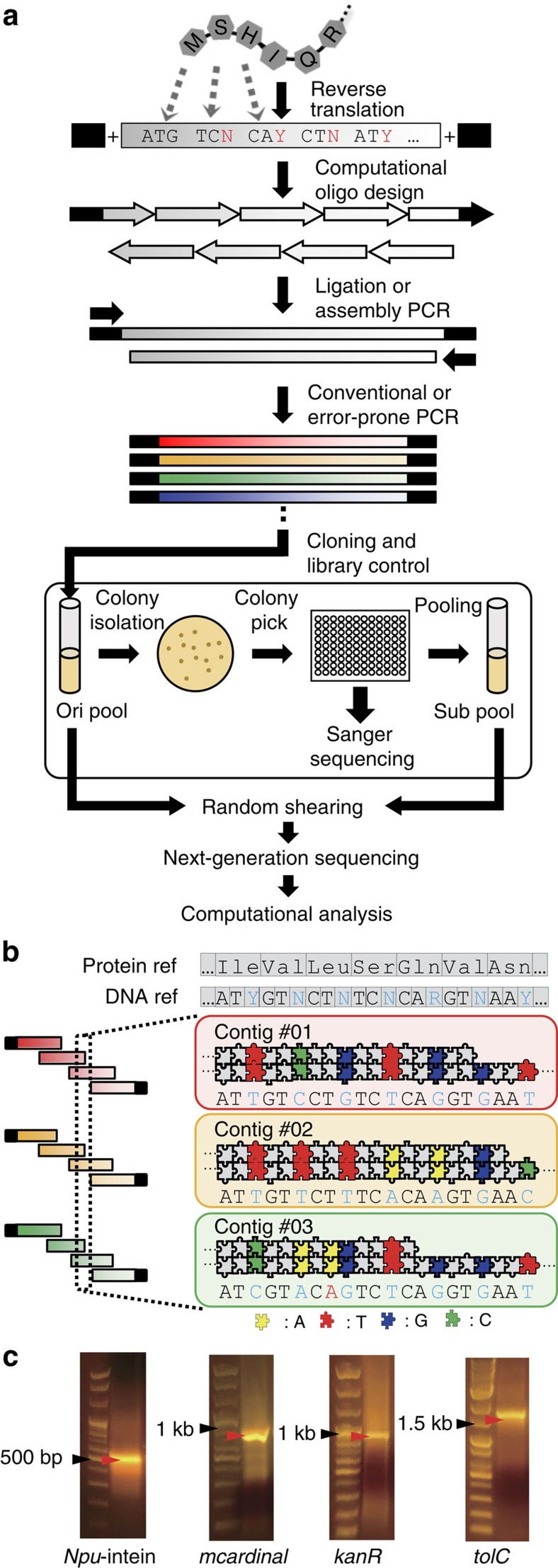
Overview of JigsawSeq pipeline. (**a**) General scheme of gene library construction leading to NGS. Native protein sequences were reverse-translated with barcodes (N, R and Y). Forward and reverse flanking sequence were attached to both ends of reverse-translated DNA sequences. Black regions represent flanking vector sequences. After LCR or assembly PCR, assembled genes were cloned into an appropriate vector. Sub-pools (Sub) were generated by isolating 96 colonies from its initial pool. Then, these pools were subjected to standard NGS library preparation. For performance validation, we verified 96 colonies by Sanger sequencing. (**b**) Schematic representation of overview of de Bruijn graph-based assembly of a codon-barcoded library. Codon-barcoded reads interlock with each other to assemble final contigs. (**c**) Library insert sizes were verified by agarose gel electrophoresis.

We constructed variant libraries for genes of various lengths (Fig. 1c): the self-splicing gene *dnaE* in *Nostoc punctiforme*^25^ (*Npu*-intein, 411 bp); the far-red fluorescent protein coding gene^26^ (*mcardinal*, 735 bp), aminoglycoside 3-phosphotransferase gene (*kanR*, 816 bp) and a transmembrane channel protein coding gene^27^ (*tolC*, 1482, bp). These genes were selected based on varying gene length for JigsawSeq efficiency modelling and their utility for protein engineering studies. In detail, the method used in construction of a library was as follows: First, we designed oligonucleotides for full-length gene assembly. The sequences were determined by reverse-translation of protein sequences with barcodes: N (for A, T, C or G), R (for A or G) and Y (for T or C). We placed N, R and Y bases at the third position on the codon (4^281^ diversity for *tolC* with reverse-translated 281 ‘N' sequences) only if no amino acid change occurs (Supplementary Table 1 and Supplementary Data 1). Incorporating degenerate nature of the genetic code, designing oligonucleotides with synonymous codons that translate into the same amino acid sequences provides diversity in making gene variant library. The unique combination of codons per one variant molecule facilitates one to deconvolute the pooled libraries by assembling consensus sequences of fragmented reads (Supplementary Fig. 1). Second, synonymous gene libraries were generated by assembly PCR^28^ or ligase chain reaction^29^ (LCR; that is, ligation reaction followed by PCR) with corresponding sense and nonsense oligonucleotides. To achieve a diverse mutational spectrum at the protein coding level, we optionally used error-prone PCR to generate libraries containing non-synonymous mutations (Supplementary Table 2 and Methods).

We then cloned the synthetic gene variants into appropriate plasmid vectors (Supplementary Figs. 2 and 3; Supplementary Table 2) and transformed them into *Escherichia coli* cells. We limited the initial population to a few thousand clones (Supplementary Fig. 4) for a model study. For a positive control, we randomly sub-sampled 96 colonies from the initial pool and combined them for subsequent NGS analysis. In addition, each colony was separately sequenced for Sanger sequencing to identify true positive sequences. For simplicity, we characterized each library with the corresponding notations; for example, *kanR*_initial is the original pool of thousands of *kanR* gene variants, and *kanR*_Sub contains the pooled 96 clones prepared from *kanR*_initial. We noted that not all colonies contained a cloned gene because of incomplete cloning efficiency. Plasmids from two different pools were randomly sheared into 200–400 bp fragments and further treated to prepare standard NGS sequencing libraries. Finally, each gene library was sequenced with the Illumina HiSeq 2500 platform (Methods).

### The core algorithm of JigsawSeq

**Figure 2:**
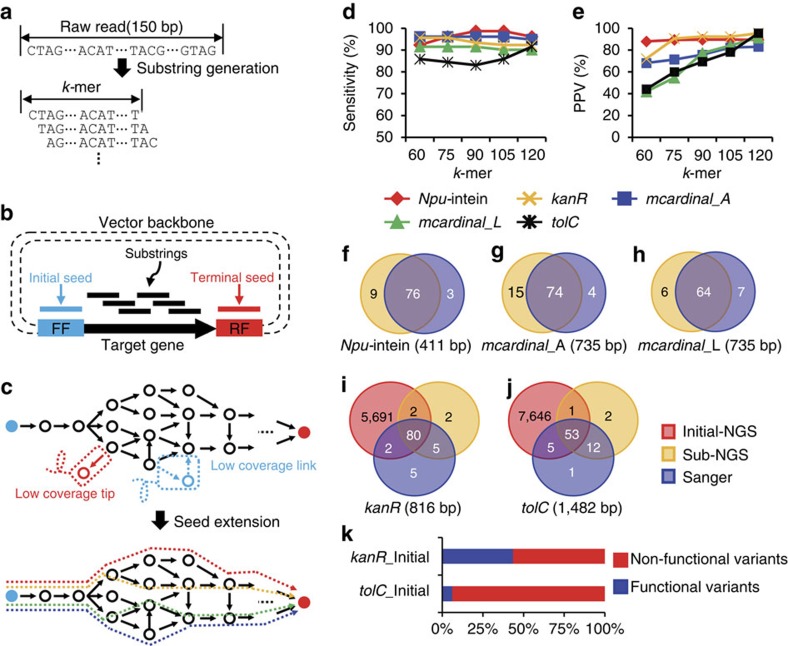
General schematic representation of workflow and performance analysis of the JigsawSeq pipeline. (**a**) Raw reads were divided into substrings. (**b**) All substrings were aligned to a vector backbone, and substrings aligned to either end (FF: forward flanking or RF: reverse flanking) of the vector backbone adjacent to the target gene were considered initial/terminal seeds. (**c**) Erroneous links (dotted blue) and tips (dotted red) were removed to reduce memory usage and improve search speed. From each initial seed (blue circle), all possible contigs were explored starting from initial seed to terminal seed (red circle). To evaluate our method, we calculated (**d**) the recall (true positive contigs called by JigsawSeq divided by all true positive contigs validated by Sanger) and (**e**) the precision (true positive contigs called by JigsawSeq divided by the assembled total contigs with different *k*-mer sizes by comparing validated Sub Sanger and Sub NGS pools. For example, *mcardinal*_L is a pool constructed from LCR (L), and *mcardinal*_A was constructed from assembly PCR (A). Comparisons between Sanger and NGS Sub-pools of (**f**) *Npu*-intein, (**g**) *mcardinal*_A and (**h**) *mcardinal*_L are shown. Overlapping contigs between initial and Sub-pools of (**i**) *kan*R and (**j**) *tolC*. (**k**) Bar plot showing distribution of initial gene variant library of *kanR* and *tolC*.

We performed *de novo* assembly of sequenced short-read data using a modified de Bruijn graph (Fig. 2a–c) to reconstruct full-length gene variants. In general, de Bruijn graph provides overlap information in DNA substrings from sequencing reads. The words of length *k* (*k*-mer) represent nodes and adjacent *k*-mers overlap by (*k*-1) mers are called edges. Assembling reads can be formulated as finding a trail or Eulerian path that visits edge exactly once. Initially, we made nodes for every (*k*-3) mer appearing as consecutive substrings of raw reads. Then, nodes were connected using edges (*k*-mer substrings of raw read) when two (*k*-3) mer nodes are the prefix and suffix of a *k*-mer edge. To save the computational costs on assembly, infrequent nodes and edges were masked according to input threshold parameters. Next, we defined the nodes aligned at the beginning and end of backbone vector sequences, adjacent to inserted gene sequence, as initial and terminal seeds. We further defined ‘contig' as the full-length DNA segment by traversing overlapped consensus nodes from initial to terminal seeds and explored all possible contigs using de Bruijn graph. Lastly, to filter spurious contigs, raw sequencing reads were re-aligned into candidate contigs, and depth distributions with exact sequence matches were examined. We assumed the read depth mapped to true positive candidate gene contigs would be evenly distributed because we randomly sheared whole plasmids containing the gene variant library. With regard to contigs of various genes confirmed by Sanger sequencing, we determined the cut-off of the coefficient of variation (CV) (ratio of the standard deviation to the mean of the aligned depth distribution), which maximizes sensitivity (recall) and predictive positive value (precision). We retained contigs with uniform depth distribution, which had a lower CV than threshold for further analysis (Methods).

### Validation of JigsawSeq on sub pool data

To optimize parameters for *de novo* assembly, we first compared Sub-NGS libraries with verified Sanger sequences and calculated precision and recall according to different *k*-mer sizes: 60, 75, 90, 105 and 120. As expected, precision increased as *k*-mer length increased because increasing *k*-mer length will cover more randomized bases, promoting contig uniqueness (Fig. 2d,e). From both recall and precision results, we determined the *k*-mer size as 120 and applied this value for analysis of all libraries.

We observed that, on average, 93% (90–96% range) of contigs detected by JigsawSeq (five sub-pools) were validated by Sanger sequencing (Fig. 2f–j). Next, we simulated our data to examine sensitivity via the number of raw reads (Supplementary Fig. 5). Overall, with even ∼1 million reads, Sub-pools showed high sensitivity (average: 81%). Notably, for *Npu*-intein, we recovered >92% of contigs using ∼1.0 × 10^6^ reads (∼0.3% of HiSeq 1 lane). Not all gene variants validated by Sanger sequencing were retrieved in our result of Sub NGS pool, partly due to non-uniform growth of cells containing specific plasmids (Supplementary Fig. 6); on average, 1.4 contigs (0–4 range for five sub-pools) in the Sanger set were unrepresented, even in the raw NGS read, indicating that missing contigs were not due to *de novo* assembly and were actually not sequenced.

Next, we evaluated potentially ‘functional variants', defined as in-frame contigs without mutations, such as insertions or deletions that would result in a premature stop codon (Fig. 2k and Supplementary Table 3). Notably, we identified mutations separated by long spans of identical sequences (Supplementary Fig. 7). Because of inherent synthetic oligonucleotide errors^30^ (usually deletion errors), as the number of oligonucleotides needed to synthesize the gene increases, more non-functional variants would likely be created. Next, when comparing two different library construction methods (for example, assembly PCR versus LCR) for mcardinal genes, we observed a marginal difference in the proportion of functional variant contigs (LCR: 41%, assembly PCR: 30%).

### Simulation of scalability in terms of data and computation

To verify that our method could be reliably scaled to larger libraries, we performed *in silico* simulation of high-throughput sequence data (150 bp paired-end reads) for *tolC* and *kanR* assuming uniformly distributed sequencing error rate of 0.1% (substitution errors are dominant in Illumina platform). The simulated templates consisted of fixed length of gene sequences plus backbone seed region (±150 bp sequences flanking the start and end of the gene) to map the initial and terminal seeds for assembly. We reasoned that removal of backbone plasmid sequences would aid accurate assembly as unwanted nodes create false paths making it computationally intensive. First, the amino acid sequence was reverse translated using N, R and Y. These three ambiguous sequences were set to one of possible bases with uniform probability. Then, random mutation was generated with various mutation rate conditions mimicking an error-prone PCR library. Finally, we simulated two different models for comparison: First, with no cell growth bias (evenly distributed number of templates of distinctive molecules) with uniform distribution of coverage. Second, coverage based on negative binomial distribution with empirical determination of parameters mu and sigma reflecting the cell growth bias observed in the initial pools (Supplementary Fig. 8). The rationale for choosing negative binomial distribution is that the model is useful for explaining biological variability.

**Figure 3:**
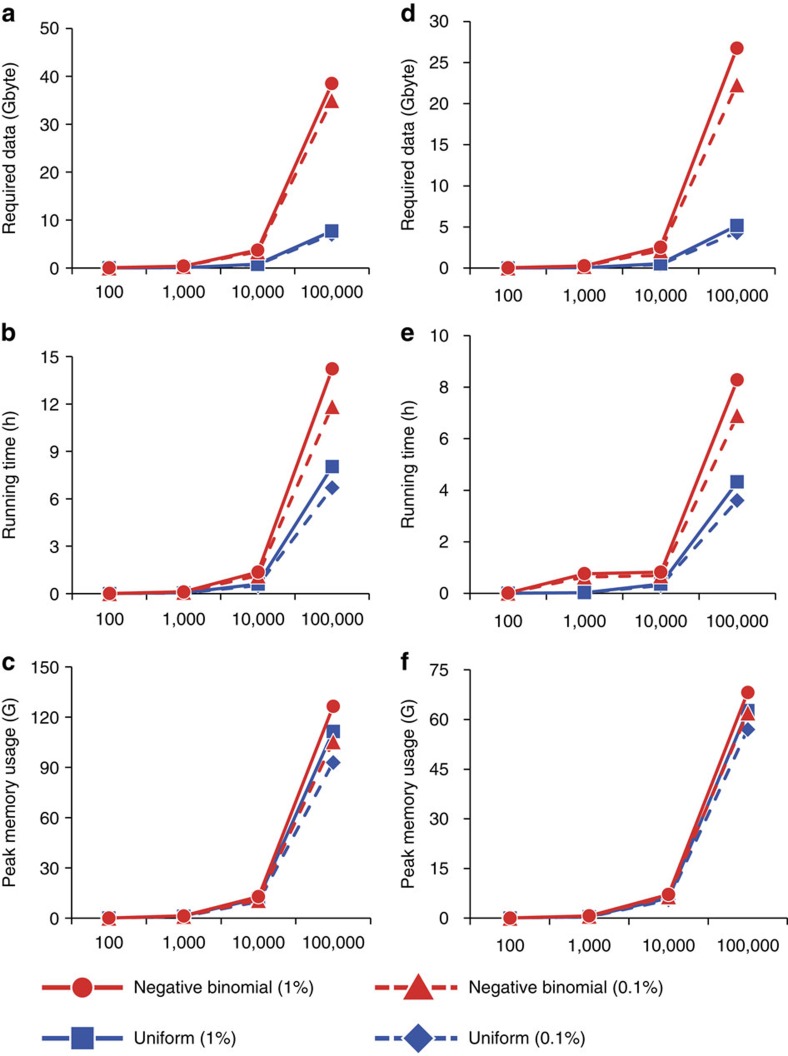
Estimating computational requirements via simulation. Random mutations were generated with specified mutation rate of 0.01 mimicking the error-prone PCR library of 100,000 *tolC* (left column). No mutations were applied for normal PCR library of *kanR* (right column) variants. Sequencing errors (substitution) were simulated for the rate of 0.1 and 1%. (**a**) The model based on uniformly distributed coverage (population) required five times less data size than the model based on negative binomially distribution. (**b**) Estimated time was 12 h and (**c**) peak memory usage of a process reached 105 GB based on negative binomial distribution. We found that memory usage between the two models were similar since it relied solely on the *k*-mer contents or *k*-mer multiplicity (proportional to the population size in the pool). For the *kanR* library (same library size as *tolC*), required (**d**) data size, (**e**) time and (**f**) memory for an assembly were lower than that of *tolC* since gene length was short (less path to traverse). Up to 10,000 variants, the effect of sequencing errors were not distinguishable (<1%) between two models. Difference in performance between two distributions suggested that even distribution of the library would reduce computational burden.

In the simulation, we found that a biased distribution of the cellular contents required higher coverage (12 × for uniform, 60 × for the negative binomial model for *tolC*, Supplementary Fig. 9 and Supplementary Fig. 10a) to rescue a rare (0.1–0.2% allele frequency) population as observed in the actual distribution of initial pools. We observed a linear increase in peak memory usage and running time with respect to the number of population to be analysed. Based on the negative binomial distribution, which reflects more realistic cell behaviour, a 10,000 variant library of *tolC* can be efficiently assembled in 1.1 h (11 G RAM with 3.3 GB data, for *kanR:* 0.7 h, 6.5 G RAM with 2.1 GB data) with high sensitivity (Fig. 3). When comparing the actual initial pool of ∼10,000 variant population with the simulated pool, we estimated that real data showed increase in running time and memory usage since we utilized sequencing reads from whole plasmid sequences, which could generate unwanted nodes (*k*-3 mer nodes created by backbone sequences) that induce ambiguous paths complicating assembly. Thus, we discarded reads that were properly aligned to the reference backbone plasmid sequences (except ±150 bp for the initial and terminal seed detection regions) to achieve more efficient performance (Supplementary Table 4).

The discrepancy between the real and simulated data results from accumulated sequencing errors as data size increases. Also, variable indel polymorphisms could be generated during the gene library construction and cloning step which also complicate graph structures. These issues together would be likely to create false nodes that hamper accurate assembly. As *k*-mer content increases (longer path), it dramatically affects memory usage and operation time. We finally demonstrated the capability to analyse 100,000 variant populations in less than 12 h (105 G RAM for *tolC)*. The whole process was performed using an Intel(R) Xeon(R) processor with a 2.93 GHz CPU with 192 GB of main memory.

### Analysis of initial pools and data estimation

When comparing contigs in the initial pool to Sanger validated sequences, we observed that 19 out of 71 true contigs were missed (14/92, for *kanR*). Functional variants from the initial pool accounted for 43.5% of the total contigs for the *kanR* library, whereas they accounted for only 6.2% of total contigs in the *tolC* library, consistent with an expected accumulation of oligonucleotide errors as gene length increases. We then investigated whether missing Sanger sequences in the initial pool were due to the parameter optimization or an insufficient amount of sequencing data. With exhaustive simulation under various parameters (Supplementary Table 5), we found that the default seed cut-off parameter was too strict for evaluating the initial pool, especially for current large pools that showed a right-skewed distribution. The default seed cut-off was determined as follows: First, the distinctive initial and terminal nodes were enumerated by aligning *k*-3 mer nodes in the graph. During the cloning step, errors might be introduced in the region that overlaps seed detection regions flanking gene sequences. Thus, given the node distribution, we filtered rare candidate seeds to discriminate erroneous seeds (by default, <1/150 of the maximum node depth). Considering the depth (we defined this value to reflect a certain cell population or plasmid copy number) distribution of the initial large pool, applying a strict seed cut-off value might have caused us to miss true contigs. This finding suggests that true contigs could be missed with the default seed cut-off because certain paths (nodes) of the graph (especially, start and end regions) would be missed by discarding the rare nodes (Supplementary Fig. 11a).

For *tolC,* out of 19 missing contigs, four were rescued when the cut-off was adjusted to 200 indicating that rare mutations were present in the flanking gene regions. For *kanR,* only 1 of the 14 missing contigs was rescued after this adjustment. Then, we additionally examined the rest of the missing contigs for the two initial libraries. We plotted the number of k-3 mer nodes (*x* axis) present in the raw fastq by sliding 3 bases as we traverse the graph for assembly (Supplementary Fig. 11b). Notably, we discovered that the rest of the missing contigs in the *kanR* and *tolC* initial pool were because of the empty nodes that were present in the middle of the graph prohibiting the full assembly. For this reason, we concluded that more sequencing would resolve the issue of missing nodes in the graph. We sequenced additional initial pool of *tolC* (30 GB of fastq, total 65 GB) to see if how many of the missing contigs could be recovered. In total, six more true contigs (rescued four additional contigs containing rare seeds and two additional contigs with empty nodes in the middle of the graph) were recovered. For *kanR,* total of four contigs were recovered (rescued one contig missed by seed cut-off and three contigs containing empty nodes) with the same amount of data as *tolC*. Remaining contigs still contained broken nodes that hampered assembly of the full-length contigs. We hypothesized that the sequencing was still insufficient to recover whole population.

To fully explore how much sequencing is required to analyse the initial pool library, we first modelled the coverage distribution to be negative binomial as described above, with a fixed mutation rate 0.01 for the error-prone library *tolC* and no mutations for normal PCR library *kanR*. We simulated virtual templates of comparable library size with actual initial pools under different mean coverages (*μ*=12, 24, 36, 48, 60). For 60x mean coverage, the recovery rate reached to 100%. Next, we sub-sampled the initial data pool to ½ fraction and plotted this against mean coverage (Supplementary Fig. 10b,c). Interestingly, the recovery rate (79%) under 36 × mean coverage in the simulation was very close to the value (81.7%) achieved under actual initial pool *tolC* data (mean coverage value: 36.8 × ). Finally, we linearly extrapolated the graph and estimated that a factor of 1.6 would ensure 60 × mean coverage. We concluded that 104 GB of sequenced data (65 GB*1.6) would saturate the larger pool (0.013 GB per variant, total data/assembled number of contigs). As in the simulation settings, discarding reads that are aligned to reference backbone plasmid sequences (except ±150 bp for the initial and terminal seed detection regions) required much less data. Thus, we recommend potential practitioner to sequence only region of interest for efficient performance. With less data size, one can analyse a large library very efficiently since unwanted backbone sequences are removed prior to the assembly step. In the case of *kanR* (Supplementary Fig. 12), the recovery rate reached 100% when coverage is 48 × . The recovery rate of *kanR* (86%) in the simulation (36 × ) was comparable to the value (89.1%) with given coverage (37.1 × ) at a factor of 1.0 (65 GB) of the actual initial data pool of *kanR.* A factor of 1.3 would ensure 48 × coverage when we extrapolated the graph. We concluded that 84.5 GB of sequenced data (65 GB*1.3) would saturate initial pool (0.014 GB per variant).

To examine the characteristics of these synthetic gene libraries for potential bias in protein expression, we calculated the codon adaptation index for functional variant contigs from the initial pools of *kanR* and *tolC*. Protein expression levels are often affected by codon usage, promoter sequences and ribosome binding sites^31^ (RBS). However, we observed codon adaptation index values converged to narrow ranges in multiple libraries (*kanR*_initial, 0.603±0.018 and *tolC*_initial, 0.608±0.013), Supplementary Fig. 13), suggesting expression artefacts would be minimized in our setting. Nonetheless, the full effects of codon usage on protein expression using our method remain to be explored, and we are actively engaging on gene-based selection system in a further study.

### Simulation under various mutagenesis scenarios

To investigate the effects of barcode density in the assembly, we sub-sampled barcodes of *tolC* under binomial distribution with parameters defined as follows (Supplementary Fig. 14); *n*=maximum possible number of barcodes, *P*=frequency of down sampled barcodes. The simulated condition was the same as described above with fixed mutation rate of 0.01 and evenly distributed coverage (12 × ). We found that downsampling to *P=*0.6 caused a significant reduction in sensitivity (<20%) when sequencing error rate is 0.1%. We then examined how fixed region (not randomized using codon) length affects the performance of a program by varying window size (Supplementary Fig. 15). As the length of fixed region is increased, we observed an increase in false positives while sensitivity remained high. Applying ∼1% of the mutation on the fixed region facilitated accurate assembly (PPV of ∼99%) since mutated sequences could serve as a unique barcode.

Furthermore, we simulated 100,000 variant library of *tolC* containing two distant regions with singleton missense mutation to explore the possibility to resolve long-range contiguity information. We randomly chose library from all possible combinations of missense mutation in two regions (20 AA (amino acids) × 34 AA position of region **A** × 20 AA × 34 AA position of region **B,** Supplementary Fig. 16). In fact, this large-scale site-directed mutagenesis can be done using microarray-programmed oligonucleotides, which could dramatically reduce cost, time and labour. We observed high sensitivity (98.2%) and specificity (97.9%) on simulation performance. Finally, we applied JigsawSeq to measure the diversity of full-length antibody sequences (Supplementary Fig. 17). We downloaded human heavy and light (kappa) chain V, D, J segment sequences from the International Immunogenetics Information System. For heavy chain, *in silico* joining of the V, D and J segments was performed. Likewise, light chain segments of V and J were also joined. The complementarity determining regions (CDRs) of single-chain antibodies are defined using Kabat's numbering scheme^32^. Heavy and light chain sequences were joined with the help of linker peptide sequences (GGSGGSGGASGAGSGGG) which were reverse translated to DNA sequences using ‘N'. Using codon redundancy, other framework regions (FRs) with the exception of CDRs were randomized with ‘N' using DP-47 and DPK-22 germline sequences^33^,^34^ which are frequently found in human antibodies. We compared performances of the assembly between randomized and non-randomized library design. We note that codon barcodes offer a great advantage in high specificity (99% versus 54%).

## Discussions

In this work, we successfully extend our method to explore JigsawSeq-based analysis of various synthetic gene variant libraries of initial pools and find high concordance with corresponding sub-NGS pools. When comparing between real and simulated data, accurate assembly can be achieved, provided that deep and uniform coverage of the population can be obtained. Although the quest for more accurate and efficient assembly software remains area of critical development, we envision that hybrid *de novo* assembly, using combination of long-read technology and short-read data offers an alternative solution. The following four points are notable for further discussion.

The originality of our method is proven based on the following four aspects. First, the simplicity of the barcoding design (the codon is randomized to ‘N','R' and ‘Y' for every third position) allows us to reassemble a large number of fragmented sequencing reads to retrace the original molecule sequences. To investigate how the barcoding density affects assembly, we randomly sampled the number of barcodes under a binomial distribution. Clearly, as decreasing the number of barcodes shows decrease in sensitivity, a maximum level of randomization of the library is essential in order to achieve high sensitivity and PPV. Specifically, the number of barcodes in the *k*-mer window provides distinct path for the *de novo* assembly step. For future users, conventional mutagenesis methods such as oligonucleotide assembly and programmed mutagenesis could also be applied in our experimental framework (Supplementary Fig. 18).

Second, *de novo* assembly may allow us to explore novel epistatic interactions between long-range mutations. We simulated assembly of random mutagenesis library containing two regions having singleton missense mutation events that are distant (>800 bp). Simulation results for potential applications of our method have proven the reliability of this method for analysing variants that lay farther apart. Further, *in silico* analysis of 100,000 unique combinations of single-chain Fv antibody libraries was carried out and a large repertoire of these antibody sequences was successfully distinguished at the single molecule level. When compared with the design with fixed nucleotide sequences in the FRs, introducing codon barcodes in the regions facilitated accurate assembly by reducing false positives significantly. The ability to identify single molecule sequences in a multiplexed fashion would shed light on the antibody engineering.

Third, we were able to utilize the sequencing depth information of each variant to successfully quantify distinctive molecules. The depth profile of each variant would provide useful metric for prioritizing variants with desired properties in selection experiments. In this study, we observed that the functional variants were highly enriched and suggested that the functionally important clones would show a high enrichment ratio (Supplementary Fig. 19). This should be validated further via additional *in vitro* protein functionality studies (e.g., enzymatic activity).

Lastly, JigsawSeq enables ‘selective retrieval' of target genes from the reassembled libraries. Using 22–24 bp of primers (including 7 bp unique nucleotides on average) at both ends of the target gene as ‘codon-based barcode tags' for selective retrieval of desired gene variants from the gene library, the recovery of the target gene sequences was successful and sequences were validated by Sanger sequencing (Supplementary Fig. 20 and Supplementary Table 6).

In summary, our results demonstrate the utility of short-read, NGS-based identification of full-length gene variant libraries in a high-throughput manner. We provide robust protocols for the construction of synthetic and codon-barcoded gene libraries. The codon-barcoding strategy highly facilitates the analysis of pooled gene variants using sequencing information obtained from randomly sheared gene fragments. Shearing whole plasmids, rather than specifically amplifying the target region, minimizes the sequencing depth bias across the gene region. As we extend JigsawSeq's portfolio of applications to analysing highly diverse variant libraries, such as antibody genes, our method will provide a new perspective for understanding the functional consequences of mutations using gene variant libraries.

## Methods

### Oligonucleotide design

Protein sequences were reverse-translated in two different ways. In the first method, protein sequences were reverse-translated using N (A, T, C or G), R (A or G) and Y (T or C) for the third base of each codon to construct a synonymous library. The other reverse-translation method used only N (A, T, C or G). For example, amino acid glycine is reverse-translated to ‘GGN' as N could be one of four possible nucleotides to make a synonymous variant library. In-house Python programming was used to design sense and nonsense randomized oligonucleotide sequences with appropriate lengths and no interval gaps. Oligonucleotide sequence information is available in Supplementary Data 1. All oligonucleotides were purchased from Integrated DNA Technologies (IDT, USA).

### Gene library construction using ligase chain reaction

Oligonucleotides were diluted to a final concentration of 10 μM. A 3 μl aliquot of each sense oligonucleotide was phosphorylated with 6 μl 10 × PNK buffer and 3 μl T4 PNK (NEB, USA). The final volume was adjusted to 60 μl by adding distilled water (dH_2_O) and incubated at 37 °C overnight. Nonsense oligonucleotide pools were phosphorylated by the same protocol. After 5′ phosphorylation, 20 μl sense oligonucleotide and 20 μl nonsense oligonucleotide solutions were mixed with 5 μl 10 × Ampligase buffer, 2.5 μl Ampligase (100 U per μl, Epicentre, USA), and 2.5 μl dH_2_O. The reaction was performed under the following conditions: (1) initial denaturation at 95 °C for 3 min; (2) annealing at 95 °C with ramping at 0.1 °C s^−1^ until reaching 60 °C; (3) ligation at 60 °C for 2 h; and (4) storage at 4 °C. The reactions were then purified with a Qiagen PCR purification kit according to the manufacturer's instructions (Qiagen, USA).

For the amplification, 1 μl assembled product from the first reaction was mixed with 7 μl dH_2_O, 10 μl KAPA HiFi 2 × polymerase (Kapa BioSystems, USA), and 1 μl each forward and reverse primers (10 μM). The mixture was subjected to PCR under the following conditions: (1) 95 °C for 3 min; (2) 95 °C for 30 s; (3) 60 °C for 30 s; (4) 72 °C for 1 min with repetition of steps (2) through (4) for 20 cycles; (5) 72 °C for 10 min; and (6) 4 °C storage.

### Gene library construction through assembly PCR reaction

Sense and nonsense oligonucleotides were diluted to a final concentration of 10 μM and mixed in equal proportions. Assembly PCR reactions contained 5 μl oligonucleotide mix, 10 μl KAPA HiFi 2 × polymerase and 5 μl dH_2_O. The assembly reactions without primers were performed as follows: (1) 95 °C for 3 min; (2) 95 °C for 30 s; (3) 60 °C for 30 s with ramping 0.1 °C s^−1^; (4) 72 °C 1 min with repetition of steps (2) through (4) for 20 cycles; (5) 72 °C for 10 min; and (6) 4 °C storage.

For the amplification, 1 μl assembled product from the first reaction were mixed together with 7 μl dH_2_O, 10 μl KAPA HiFi 2x polymerase, and 1 μl each forward and reverse primers(10 μM). The mixture was subjected to PCR with the following conditions: (1) initial denaturation at 95 °C for 3 min; (2) annealing at 95 °C with ramping at 0.1 °C s^−1^ until reaching 60 °C; (3) ligation at 60 °C for 2 h; and (4) storage at 4 °C. The reactions were then purified with a Qiagen PCR purification kit according to the manufacturer's instructions (Qiagen, USA).

### Randomly-mutated gene library construction

We used the GeneMorph II Random Mutagenesis kit (Agilent Technologies, USA) for error-prone PCR. Using the assembly PCR product as a template, amplification was carried out using the error-prone PCR protocol according to the manufacturer's instructions. After mixing 1 ng (1 μl) template, 5 μl 10 × Mutazyme II reaction buffer, 1 μl 40 nM dNTP mix, 1 μl Mutazyme II and 2 μl each forward and reverse primer (10 μM). The final volume was adjusted to 50 μl by adding dH_2_O. The mixture was subjected to PCR under the following conditions: (1) 95 °C for 2 min; (2) 95 °C for 30 s; (3) 55 °C (*T*_m_ −5 °C) 30 s; (4) 72 °C (1 kb min^−1^) with repetition of steps (2) through (4) for 35 cycles; (5) 72 °C for 10 min; and (6) 4 °C storage. Elongation time varied with gene length. Gel electrophoresis was then used to selectively retrieve PCR products, which were purified with a Qiagen gel purification kit per the manufacturer's protocol.

### Construction of backbone plasmid pBR322-du1

In 2009, the Muir group reported an intein selection system using the kanamycin resistance gene. Their *kanR*-*Npu* selection plasmid includes *Nsi*l and *Nco*l sites, which were inserted into the *kanR* gene through silent mutation. Intein cloning generates a split form of *N-kanR* and *C-kanR*. We obtained this plasmid from the Muir group and retrieved the *kanR* sequences from its RBS to the terminator using PCR (Forward: 5′-atcgataagcttgagcgcaacgcaattaatgt-3′, Reverse: 5′- tatagcgctagcGAATTAATTCttagaaaaactcatcgagc-3′). The retrieved sequences were cloned into a low-copy plasmid (pBR322), and the internal intein sequence was substituted by an 87 bp dummy sequence (‘5-TACAAATCCGCCTAGAGCGGATTTGAACGTTGCTGAAGCAACGGCCCGGAGGGTGGCCAGGACGGCCATTGACTGCCAGGAATTAAC-3') via *Nsi*l and *Nco*l restriction digestion to yield a control plasmid (pBR322-du1) (Supplementary Fig. 2).

### Cloning of codon variant library to pBR322-du1 or pEGFP-C1

For cloning intein, the dummy control sequence in the pBR322-du1 plasmid was replaced by intein library sequences using *Nsi*I and *Nco*I restriction sites. Gene *kanR*, which was constructed by LCR, was cloned into the pBR322 backbone plasmid using a one-step isothermal reaction (Gibson assembly^35^). Because the backbone vector is large, we divided it into two fragments with an 80 bp-overlap (Supplementary Fig. 3). The amplified gene insert and the two pBR322 backbone plasmid fragments were mixed in equal molar ratio to a final volume of 5 μl, and 15 μl assembly master mixture was added before incubation at 50 °C for 2 h. We constructed the *tolC* library using assembly PCR and cloned the inserts into the backbone vector by Gibson assembly. For cloning *mcardinal*, we first amplified gene products using both assembly PCR and LCR. We then cloned the amplicons into a pEGFP-C1 backbone plasmid using Gibson assembly and transformed into competent *E. coli* cells (C2566, NEB, USA).

### Validation of sub pool by Sanger sequencing

To test the validity of our method, we confirmed randomly-selected colonies from every gene library pool by Sanger sequencing. Primers for PCR were designed from ±50 bp upstream and downstream of the target gene. Sequence analysis was performed using Lasergene 10/SeqMan 5.01 (DNASTAR Inc., USA) (Supplementary Data 2).

### Next-generation sequencing and quality filtration

By selecting the desired number of clones, we controlled the maximum size (96 colonies for Sub-pools and up to thousands of colonies for initial pools) for our conventional and error-prone PCR libraries. The plasmids from the clone libraries were extracted using the Exprep plasmid DNA purification kit (GeneAll, Korea) and were randomly sheared to a target size of 200–400 bp using an M220 ultrasonicator (Covaris, USA). Libraries were constructed using the SPARK DNA sample preparation kit (Enzymatics, USA) according to the manufacturer's protocol. We further processed 1 μg of sheared DNA by end-repairing with end repair mix and dH_2_O up to 100 μl. Next, we mixed 50 μl of end-repaired product to A-tailing mix. Then, the Illumina adaptor loop (NEBNext Multiplex Oligonucleotides for Illumina kit; NEB) was attached to 40 μl dA-tailed DNA using ligation mix. After cutting the loop with 3 μl of the USER enzyme (New England BioLabs), we selectively purified 300–500 bp fragments through gel electrophoresis. Finally, the enrichment reaction with an index primer was carried out. We sequenced 150 bp paired-end reads with the Illumina HiSeq2500 platform. To remove sequencing errors, bases with a Phred quality score of <20 at both ends and more than three consecutive A's at the 3′-end were trimmed.

### Construction of contigs by *de novo* assembly of short reads

JigsawSeq is a directed acyclic multi-path searching graph based on *k*-mer strategy. The algorithm consisted of seven steps (Supplementary Fig. 21). (1) The trimmed reads were divided into *k*-mer substrings. (2) To efficiently handle large fastq data, we divided these *k*-mer substrings into certain number of bins. A modified de Bruijn graph was constructed for each bin using (*k*-3) mer nodes and *k*-mer edges. The depth of nodes and edges was defined as the number of detected nodes and edges from raw reads. (3) The graphs constructed in each bins were merged into single file. For efficient memory usage, we utilized an indexing strategy using an alphabetically sorted hash table. (4) To save time and memory usage in the reconstruction process via the de Bruijn graph, any nodes or edges that appeared only once in the graph were considered errors and were eliminated. In addition, for each node, the edge with the highest depth was selected, and edges were pruned if the edge had a depth of <1/50 of the highest depth linked to a node. (5) Next, all nodes were aligned to the backbone vector sequences using Burrows-Wheeler Aligner (BWA)^36^ (0.7.5a-r405) under non-default parameters (−O2 −E1). Only nodes aligned at the beginning and end of backbone vector sequences, adjacent to the inserted gene sequence, were considered initial and terminal seeds. We neglected rare seeds whose depth was <1/200 of the highest depth of seeds. (6) With passed initial and terminal seeds, and all possible candidate contigs were explored by traversing the de Bruijn graph from initial to terminal seeds. Recursive path exploration was performed until the nodes and edges from the hash table were exhausted. For every greedy extension, we calculated (*k-3*) mer nodes (coverage) for each final contigs. We then defined plasmid copy number variation as the minimum value of all traversed (*k-3*) mer nodes. (7) To remove chimeric false-positive contigs from among the candidates generated during assembly, we aligned paired-end raw reads to candidate contigs with exact matches using BWA. Then, we calculated the CV of depth distribution (the standard deviation of depth divided by the mean depth) for each candidate. We excluded candidates with a higher CV than the cut-off (0.2163 for *k*=120), which was determined through optimization with validated contigs via Sanger sequencing. In the algorithm optimization process, we found that aligning raw fastq reads using BWA requires a high memory server and is also time-consuming which allowed mild gain to occur in PPV. Thus, this final filtering step is optional for users. Finally, contigs were aligned to the reference sequence by implementing a Needleman–Wunsch algorithm in the BioPython function (match score=2, mismatch score=−1, gap initiation=−3 and gap propagation=−1) to detect substitutions and indels.

### Retrieval of specific variants of *kanR* variant library

Primer design - To achieve an appropriate retrieval rate, 5′ and 3′-end of the primer were designed to target randomized base, specifically, the forward primer is 24 bp including the start codon of *kanR* and the reverse primer contains 22 bp sequences including the stop codon. On average, seven randomized bases (‘N','R','Y') were included in the forward and reverse primer regions.

PCR amplification—PCR reactions contained 10 ng of *kanR* plasmid pool, 10 μl of KAPA HiFi 2 × polymerase, and 1 μl of 10 μM forward and reverse primers. The conditions for PCR were as follows: (1) 3 min of initial heating at 98 °C followed by 40 cycles of 98 °C 30 s, 65 °C 30 s, 72 °C 1 min and a final elongation step at 72 °C for 10 min. Bands at 816 bp were excised from the agarose gel and purified using a gel purification kit (Qiagen) and the purified DNA was subjected to Sanger sequencing.

### Positive selection for *kan*R with kanamycin selection

We performed genetic selection based on kanamycin resistance to screen enriched population for *kanR* library. First, 100 μl of initial pool was transferred to 10 ml conical tube containing LB/Amp (50 μg per ml) and kanamycin (100 μM), then was incubated for 16 h at 37 °C. We hypothesized that significant proportion of non-functional dead clone would be present in solution after just one round of selection. Therefore, we transferred 100 μl of the previous selected population to the 10 ml conical tube containing LB/Amp (50 μg per ml) and kanamycin (100 μM). Plasmid DNAs were extracted from cloned libraries and randomly sheared with ultrasonicator to a target size of 200–400 bp. Libraries were constructed using the SPARK DNA sample preparation kit (Enzymatics, USA) according to the manufacturer's protocol.

**Availability**

JigsawSeq (version r3) is currently written in Perl and is freely available as open-source software at https://sites.google.com/site/duheebanglab/software/jigsawseq. It is released under a CC BY-NC-SA license.

## Additional information

**Accession codes**: Raw sequencing data are available under Sequence Read Archive: SRP050292 (HiSeq data).

**How to cite this article:** Cho, N. *et al.* *De novo* assembly and next-generation sequencing to analyse full-length gene variants from codon-barcoded libraries. *Nat. Commun.* 6:8351 doi: 10.1038/ncomms9351 (2015).

## Supplementary Material

Supplementary Information Supplementary Figures 1-21 and Supplementary Tables 1-6

Supplementary Data 1 Reverse translation and oligonucleotide design of target genes

Supplementary Data 2 Sanger-validated sequences
