# Supplementary material for: De novo assembly and next-generation sequencing to analyse full-length gene variants from codon-barcoded libraries
Source: Nat Commun. 2015 Sep 21;6:8351. doi: 10.1038/ncomms9351 (PMC4595759; doi:10.1038/ncomms9351)
Supplement: Supplementary Information — Supplementary Figures 1-21 and Supplementary Tables 1-6 [file ncomms9351-s1.pdf]

Supplementary Figures

Supplementary Figure 1. Schematic representation of the JigsawSeq algorithm

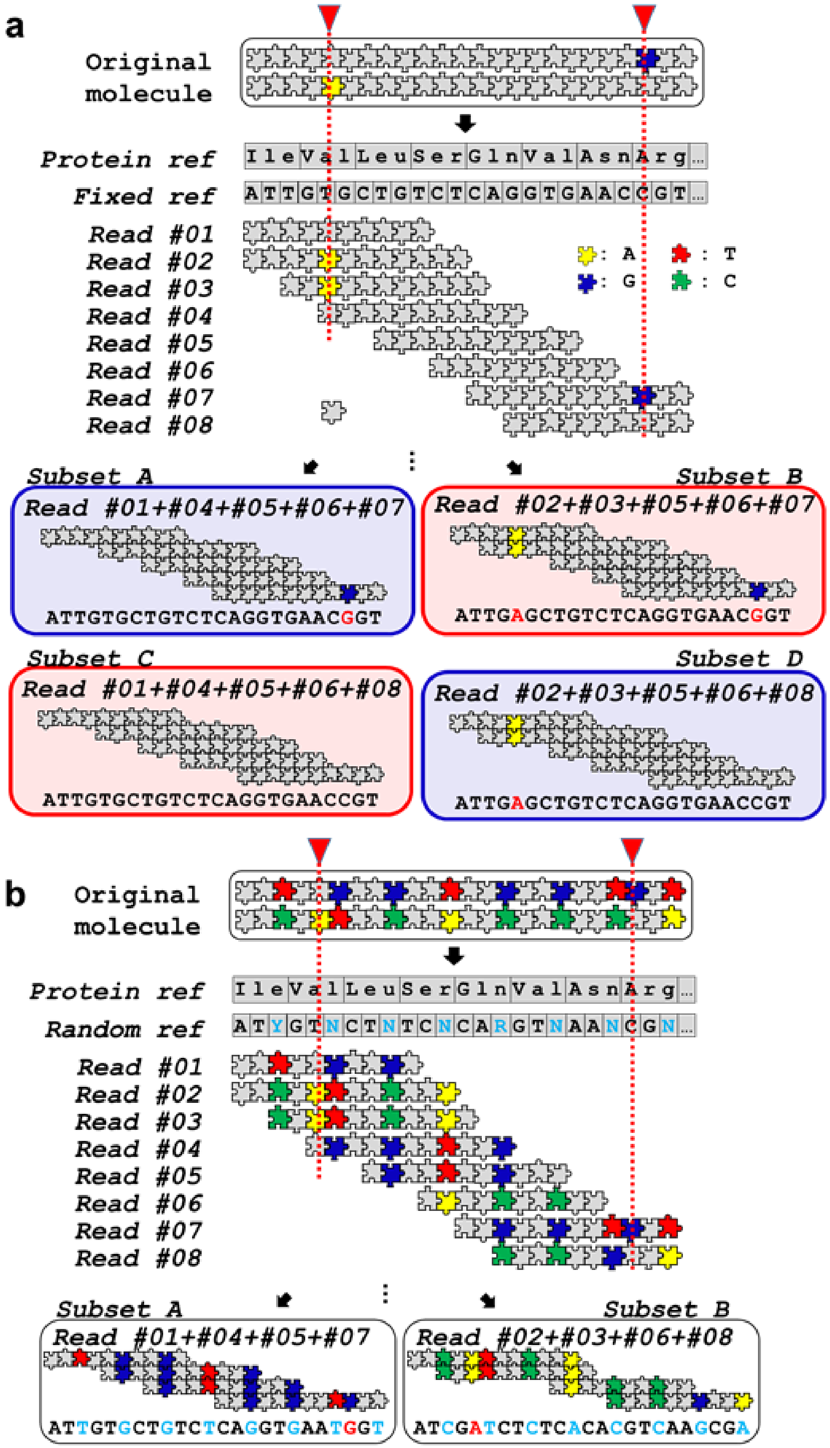

When analyzing highly homologous library (gray region) with short-read length (i.e., Illumina <150 bp) sequencer, without codon barcode (a), assembling reads results in four possible combinations. The red dotted line represents sequencing errors. Two (subset A, D) were correctly called. However, utilization of codon barcodes enables specific assembly (b).

Supplementary Figure 2. Construction of pBR322-du1 plasmid

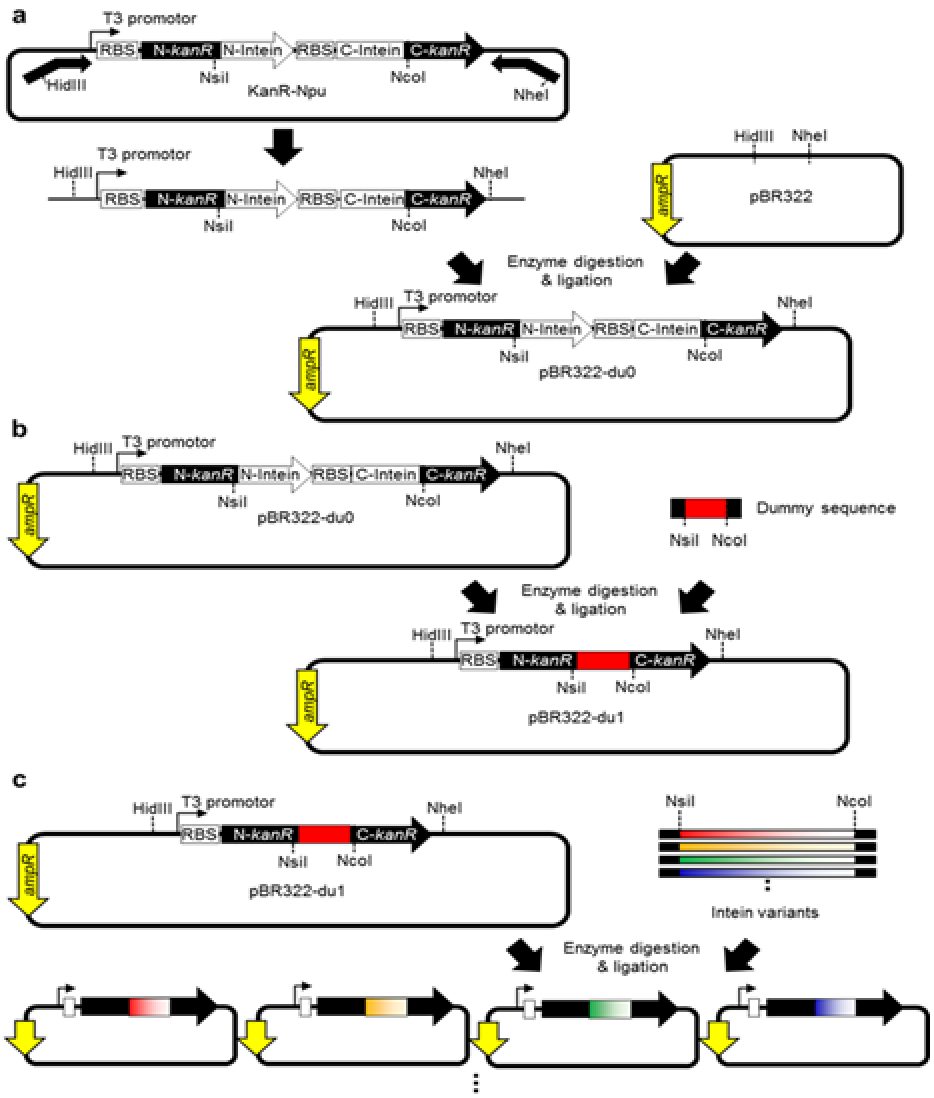

(a) Using the forward primer containing a *Hind*III site and the reverse primer containing an *Nhe*I site, we amplified the regions beginning with the promoter to the open reading frame of C-*kanR*. (b) Using *Hind*III and *Nhe*I enzymes, we cloned the fragment into pBR322 to construct pBR322-du0. We then constructed the pBR322-du1 plasmid by substituting the intein sequence with an 87-bp dummy sequence. (c) Finally, with enzyme *Nsi*I and *Nco*I, intein variants were cloned into a vector.

Supplementary Figure 3. Cloning of codon variant libraries through Gibson assembly.

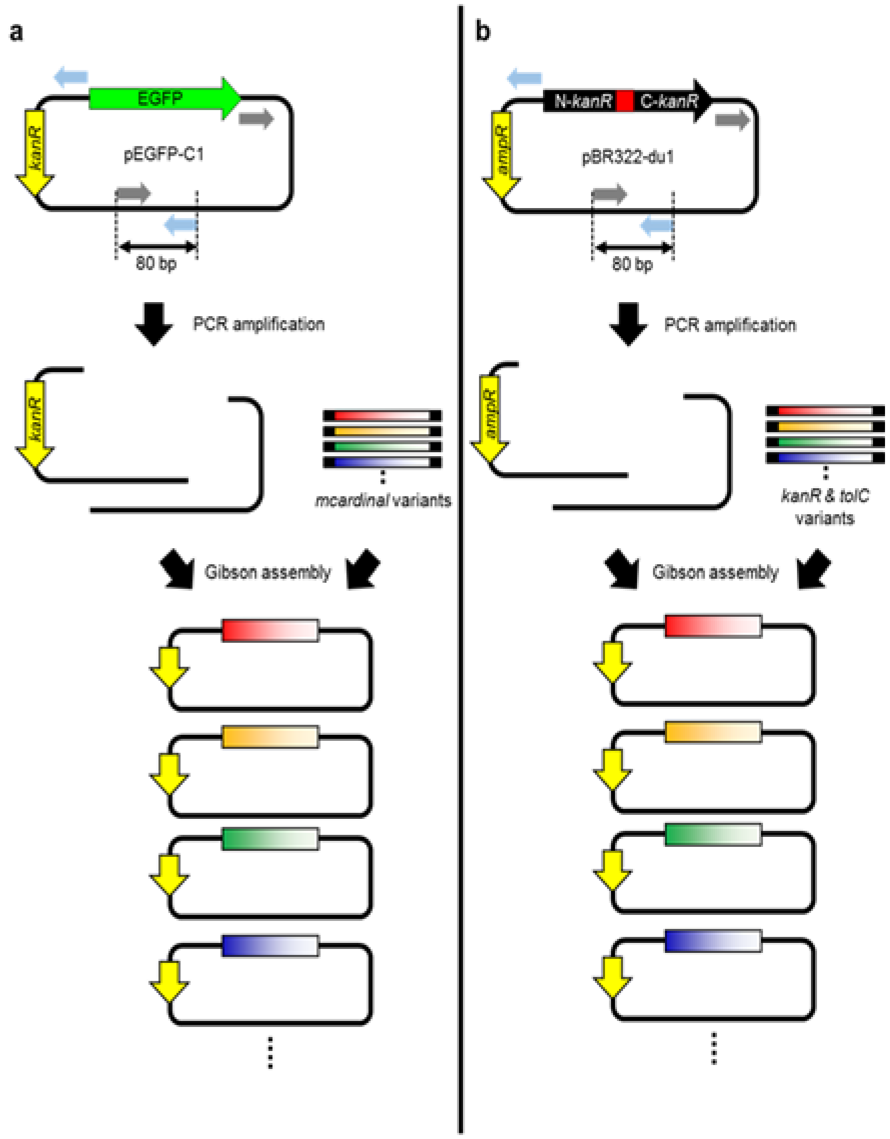

Primer pairs (blue and gray arrows) were used to amplify backbone sequences, resulting in fragments of similar lengths. Combined with synthesized gene libraries, we cloned synthesized genes into the pEGFP-C1 (a) and pBR322-du1 (b) plasmids using Gibson assembly (Online Methods).

Supplementary Figure 4. Procedure for constructing variant library pools

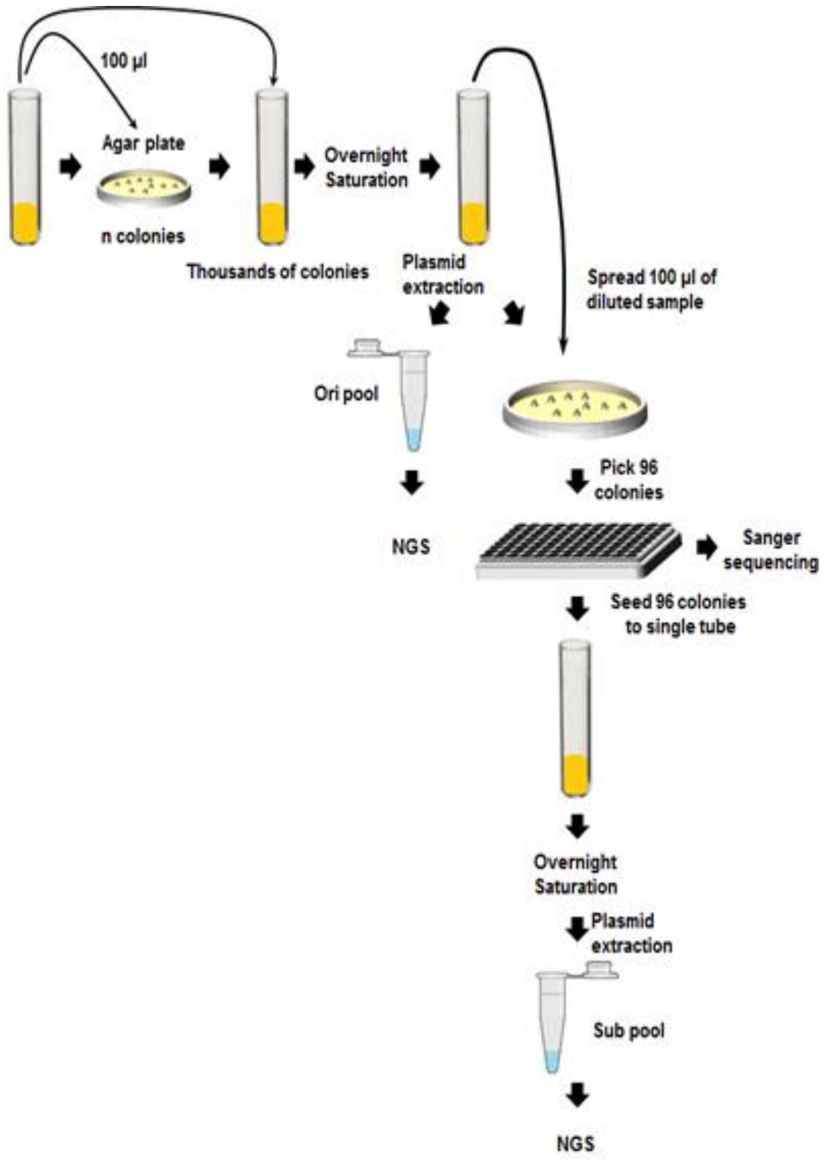

The constructed plasmids were transformed into chemically-competent *E. coli* C2566, and cells were recovered after incubating for 1 h at 37ºC. By spreading 100 µL of the initial cells, we could estimate the volume required to generate the initial pool. This pool was saturated overnight. We then extracted plasmids, and they were subjected to a standard NGS preparation protocol. The diluted pool was also spread on agar plates, from which we randomly selected 96 individual colonies. The 96 clones were mixed, saturated, and prepared for NGS as described above, and these clones represented the sub-pool (Sub) from larger initial pool of clones.

Supplementary Figure 5. Downsampling analysis for Sub Sanger pools

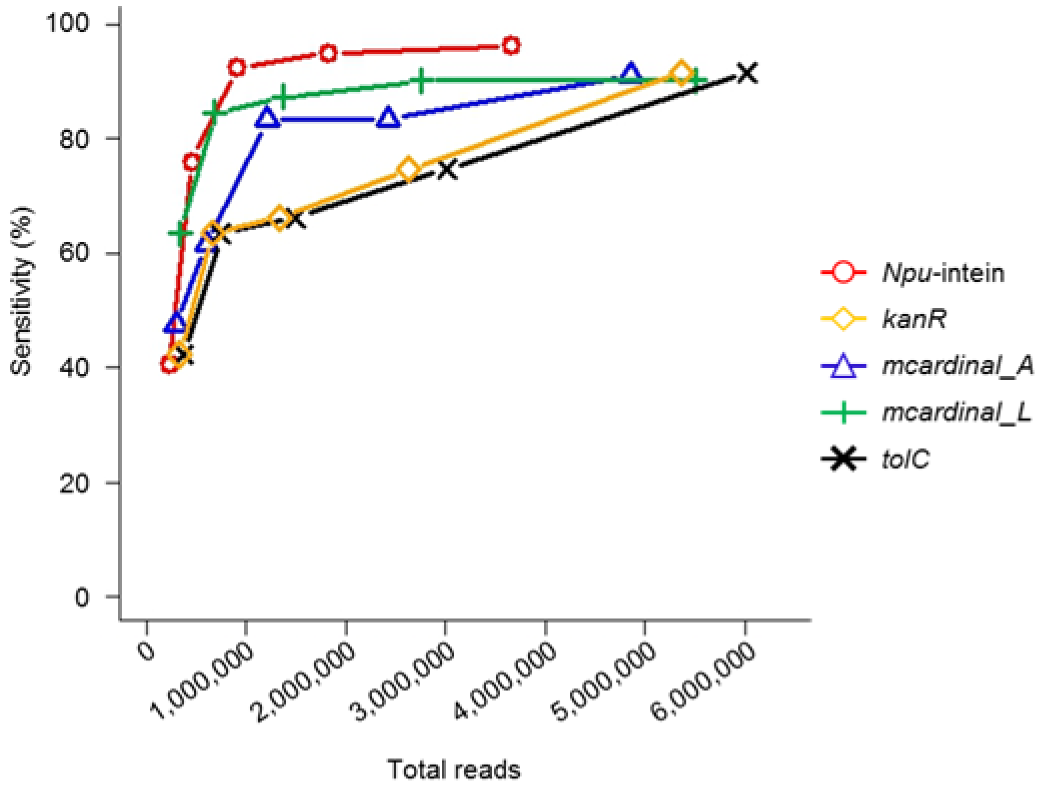

Random subsampling of the total reads (quality trimmed as described in Online Methods) for each library was performed. For *Npu*-intein and *mcardinal* genes (approximately 1.0 x 10^6 reads; 0.3% of one Illumina HiSeq lane), sensitivity remains high [(92.4% for intein (916,800 reads), 87.3% for *mcardinal*_L (LCR, 1,380,740 reads), and 83.3% for *mcardinal*_A (Assembly PCR, 1,214,230 reads)].

Supplementary Figure 6. Depth distribution of NGS Sub pools

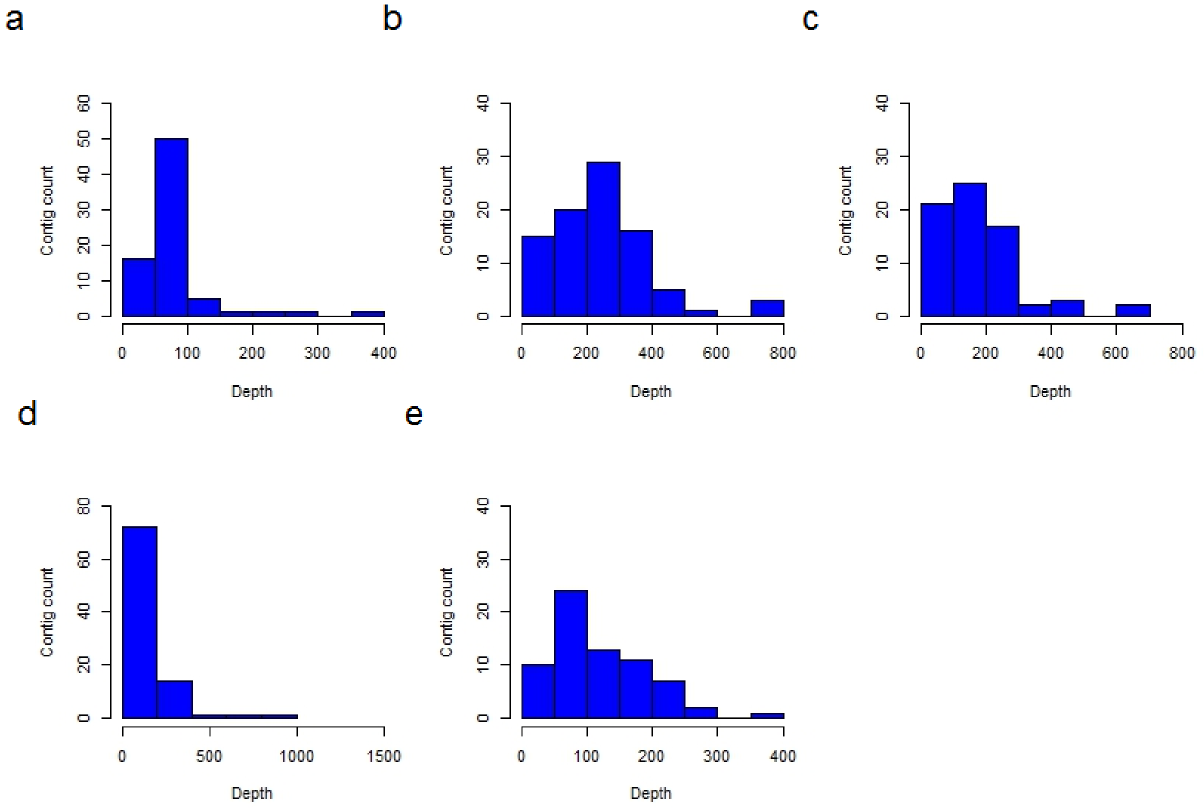

Contigs predicted by JigsawSeq and the distribution of their mean depth (variant population) in various libraries is shown: (a) *Npu*-intein_Sub, (b) *mcardinal*_A (Assembly PCR), (c) *mcardinal*_L (LCR), (d) *kanR*, (e) *tolC.* We assumed mean depth distributions of the library would reflect the variant population, and we observed amplification biases in the variant library.

Supplementary Figure 7. Alignment result of a contig from the *tolC* Sub Sanger pool

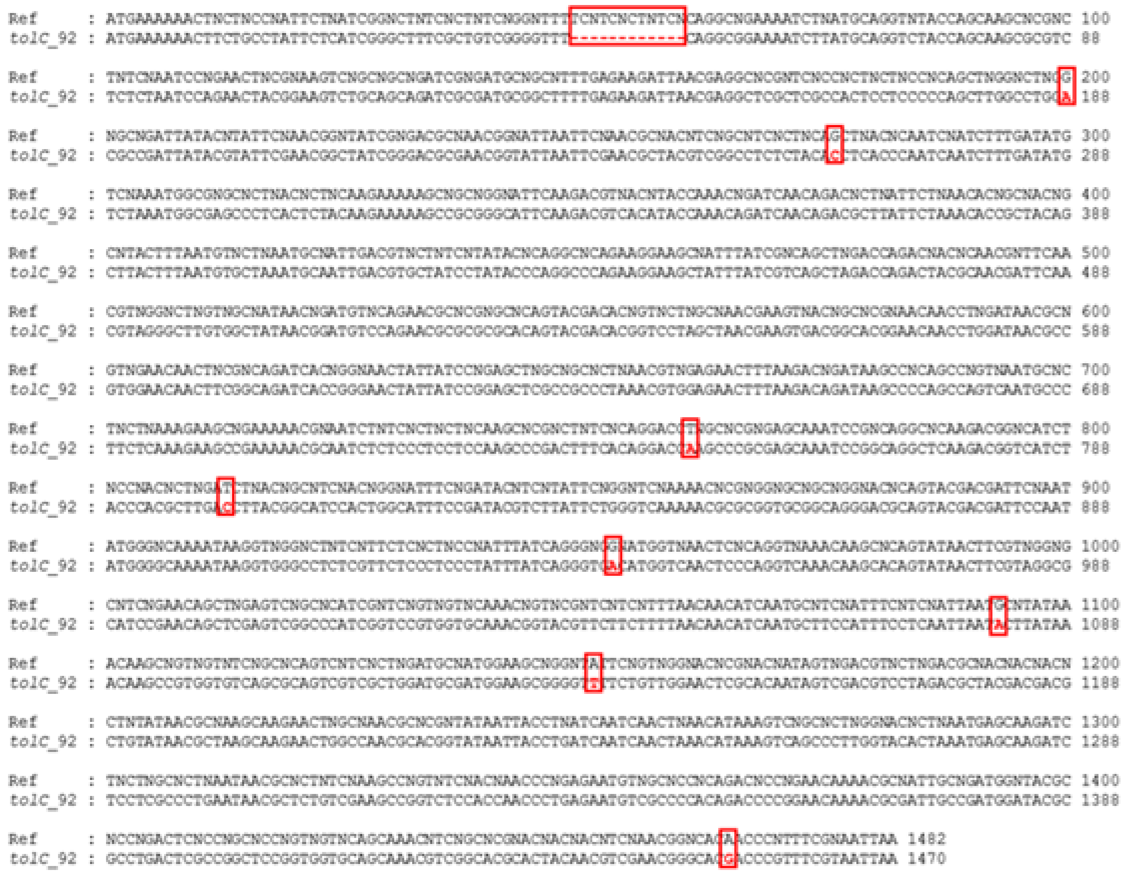

Representative contig from the Sanger-validated *tolC*_Sub pool aligned to its reference sequence. Eight substitutions and 12 consecutive in-frame deletions are shown in red box. Mutations that occur between long stretches of wild type sequence can be resolved by connecting edges of the pool's de Bruijn graph. Randomized codons, which served as barcodes, provided sufficient diversity to distinguish each variant.

Supplementary Figure 8. Depth distribution of NGS initial pools

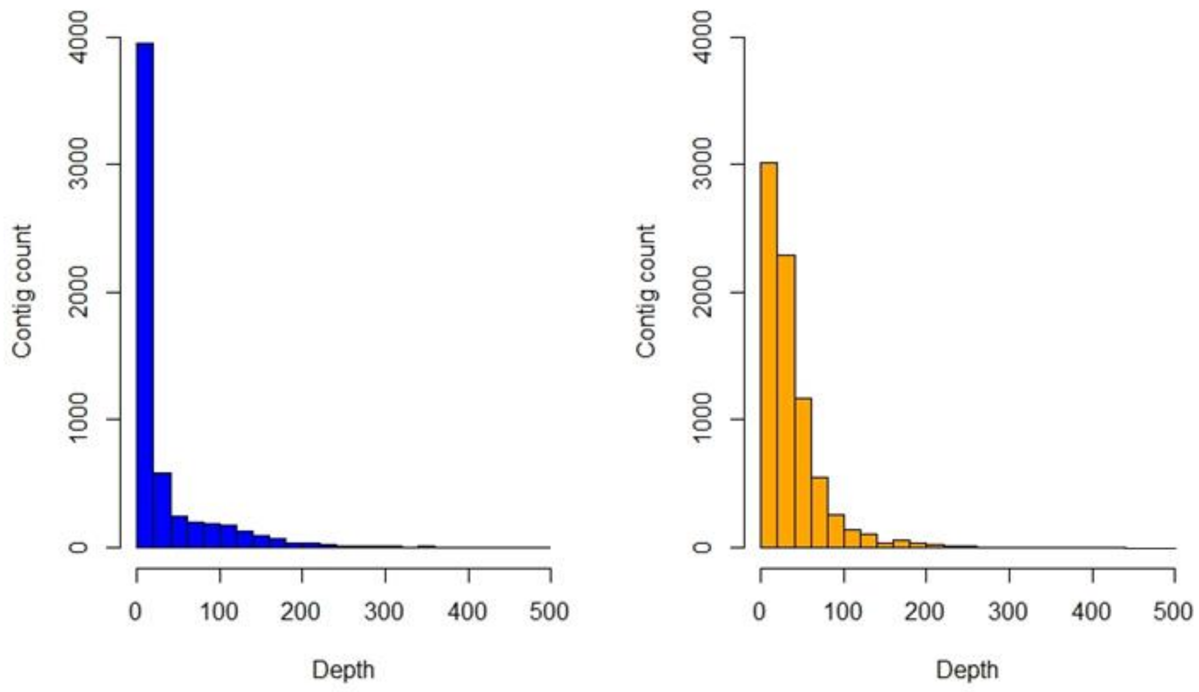

Depth distribution of the initial *kanR* (left) and *tolC* (right) pools are shown. The majority of the population distribution is concentrated on the left (positively skewed). To model over-dispersed data, negative binomial model is generally preferable. The variance-to-mean ratio (VMR, σ^2/μ) is calculated for each pool and samples drawn from this parametric distribution (negative binomial) is applied in the simulation study.

Supplementary Figure 9. JigsawSeq simulation performance

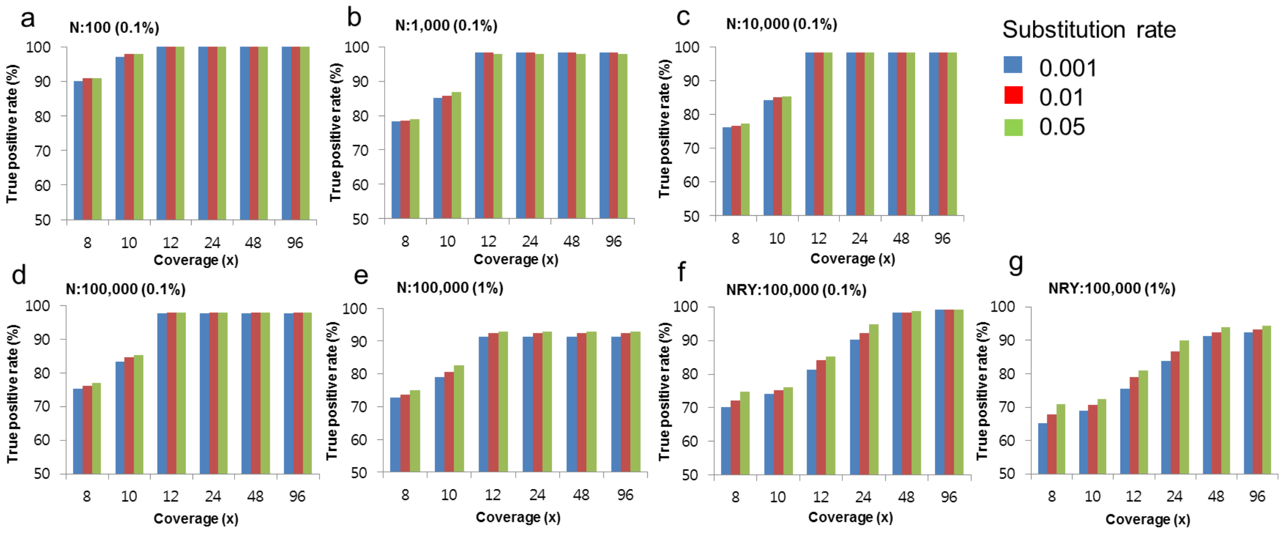

Simulation results of (a) 100, (b) 1,000, (c) 10,000, (d~f) 100,000 *tolC* mutant library populations. We set the variable as coverage (x) and mutation rate for the error-prone PCR model. Random mutations were generated with specified mutation rates of 0.001, 0.01 and 0.05. Additionally, we assumed that no amplification biases in PCR (an even number of templates of distinctive molecules) and applied sequencing error rate of 0.1% (a~d, f) and 1% (e, g). Across all levels, the recovery of template sequences was nearly perfect with reasonable mutation rate and coverage. Overall, a minimum depth of coverage value ~12 x ensures high sensitivity across all simulations. PPV for all simulation result was 100%. Additionally, we designed the *tolC* gene library with a randomization method using N, R, and Y. Since library complexity increases with an increasing number of barcodes, the coverage required to achieve comparable sensitivity increases. The sensitivity was slightly higher (98.8% vs 97.8% for 0.1% error, 93.8% vs 93% for 1% error) than that of the gene library using only ‘N’ when the depth of coverage was sufficient (> 48 x). We note that the model based on the sequencing error rate of 0.1% would reflect more realistic scenarios (the error rate of Illumina sequencer is approximately 0.1%).

Supplementary Figure 10. Data estimation for the initial *tolC* pool

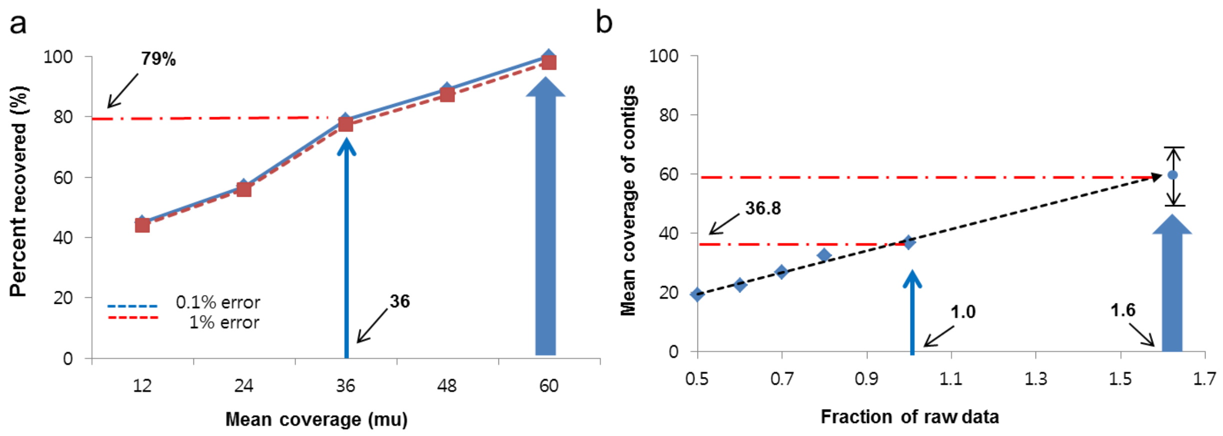

c

Factor	1.0x	0.8x	0.7x	0.6x	0.5x
Raw	65	52	45.5	39	32.5
†W / out backbone	19.6	15.7	13.8	11.8	9.8

****Factor means required scale compared to original fastq data size (1.0x)**

†Calculated after backbone-aligned reads were discarded (Gbytes, GB)

In the simulation, for 60x mean coverage, recovery of true positives was perfect (a). Even when sequencing errors are introduced at the rate of 1%, 60x ensures maximum recovery. We extrapolated the graph (b) using the R function predict.lm() and estimated the required data size (Gbytes) (95% prediction interval for a future Y observation when x=x*(1.6)). Considering the current trend of the backbone-removed data above, we expected 31.4 Gbytes (19.6 GB*1.6) would be enough to saturate a larger pool (c).

Supplementary Figure 11. Schematic diagram of the causes of missed true contigs

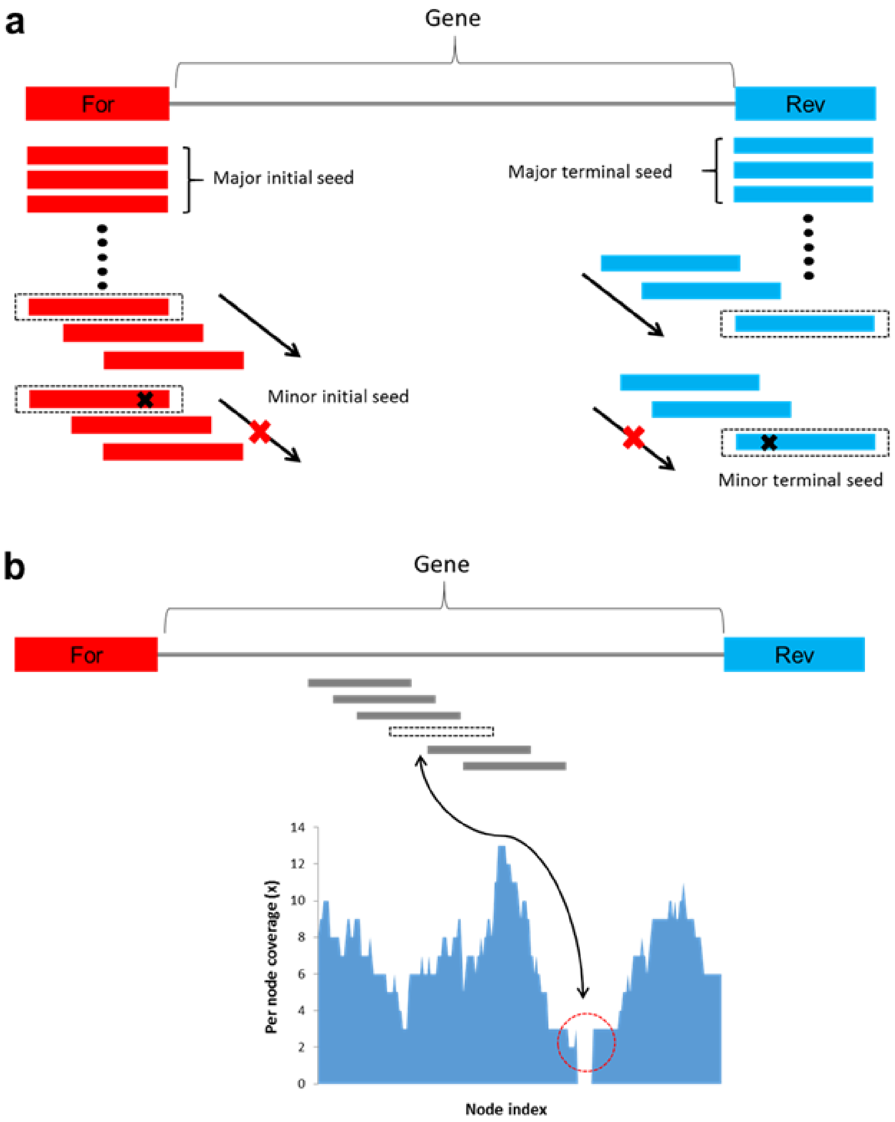

Rare seeds (a) and edges (b) could be missed using the default parameters. We could rescue these sequences by raising the cutoff value. However, erroneous edges from sequencing errors create a complex graph structure, thereby posing additional computational challenges and false assembly. Therefore, we optimized parameters for robust error correction (Online methods, edge cutoff: 50, seed cutoff: 200). In the above figure, the x-axis presents k-3 mer node position, which was defined by sliding the nodes by 3 bases from the start to the end of the gene sequences.

Supplementary Figure 12. Data estimation for the initial *kanR* pool

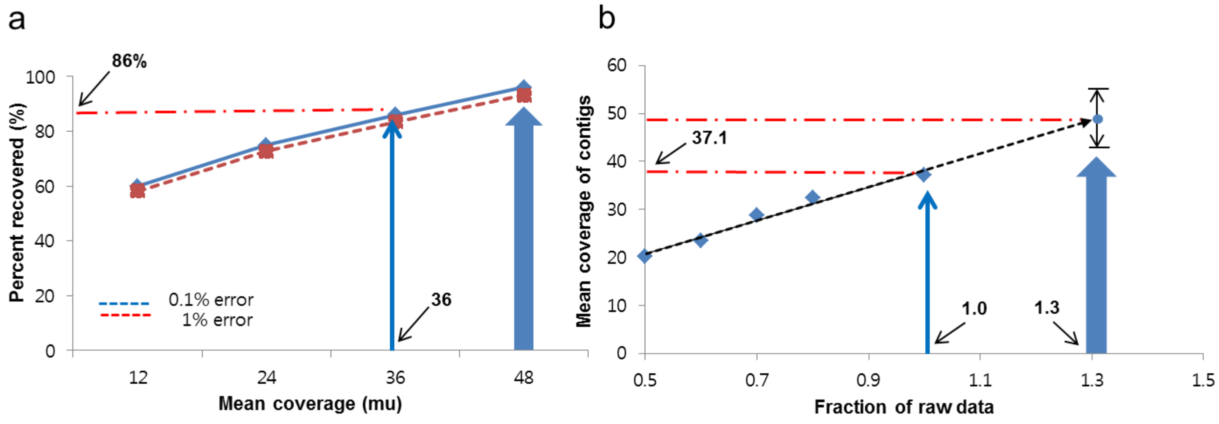

c

Factor	1.0x	0.8x	0.7x	0.6x	0.5x
Raw	65	52	45.5	39	32.5
†W / out backbone	13.8	11.1	9.7	8.3	6.9

**Factor means required scale compared to original fastq data size (1.0x)

†Calculated after backbone-aligned reads were discarded (Gbytes, GB)

In contrast with *tolC*, recovery rate reached 100% in the simulation at 48x coverage (for both 0.1% and 1% sequencing error simulations) (a). Using the same method as *tolC,* we extrapolated the graph (b) to calculate required data (Gbytes) size (95% prediction interval for a future Y observation when x=x*(1.3)) When removing vector backbone sequences, 17.9 Gbytes (13.8 GB*1.3) of data would be sufficient (c).

Supplementary Figure 13. Codon adaptation index (CAI) distribution of functional variants of *kanR* and *tolC.*

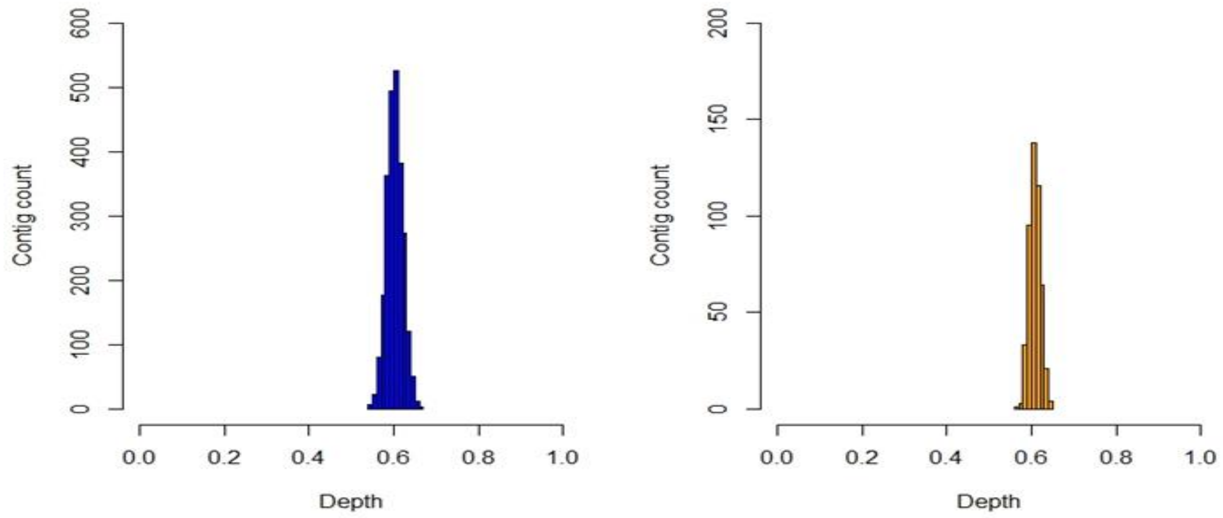

Calculated CAI distribution of both *kanR*_Ori (0.603±0.018, left) and *tolC*_Ori (0.608±0.013, right) pool variants are shown. Specifically, functional variants (in-frame contigs without premature stop codons), reveal low standard deviations.

Supplementary Figure 14. Simulation of barcode density downsampling

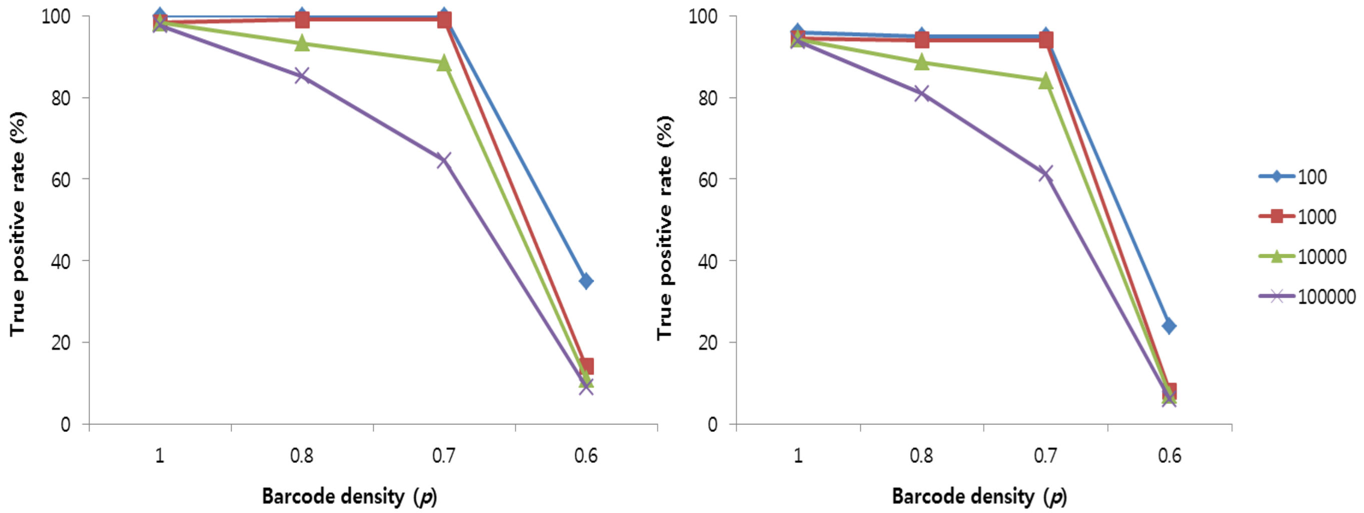

As the number of barcodes decreases, the recovery of true templates decreases (sequencing error rate of 0.1% (left) and 1% (right). As the population size increases, this effect is more pronounced. In the population up to 1,000, downsampling of barcodes to *p*=0.7 (sampling the number of barcodes in binomial distribution) showed high sensitivity. The simulation is evaluated based on optimized *k*-mer length 120.

Supplementary Figure 15. Simulation results of amino acid length variation for which translated into fixed DNA sequences

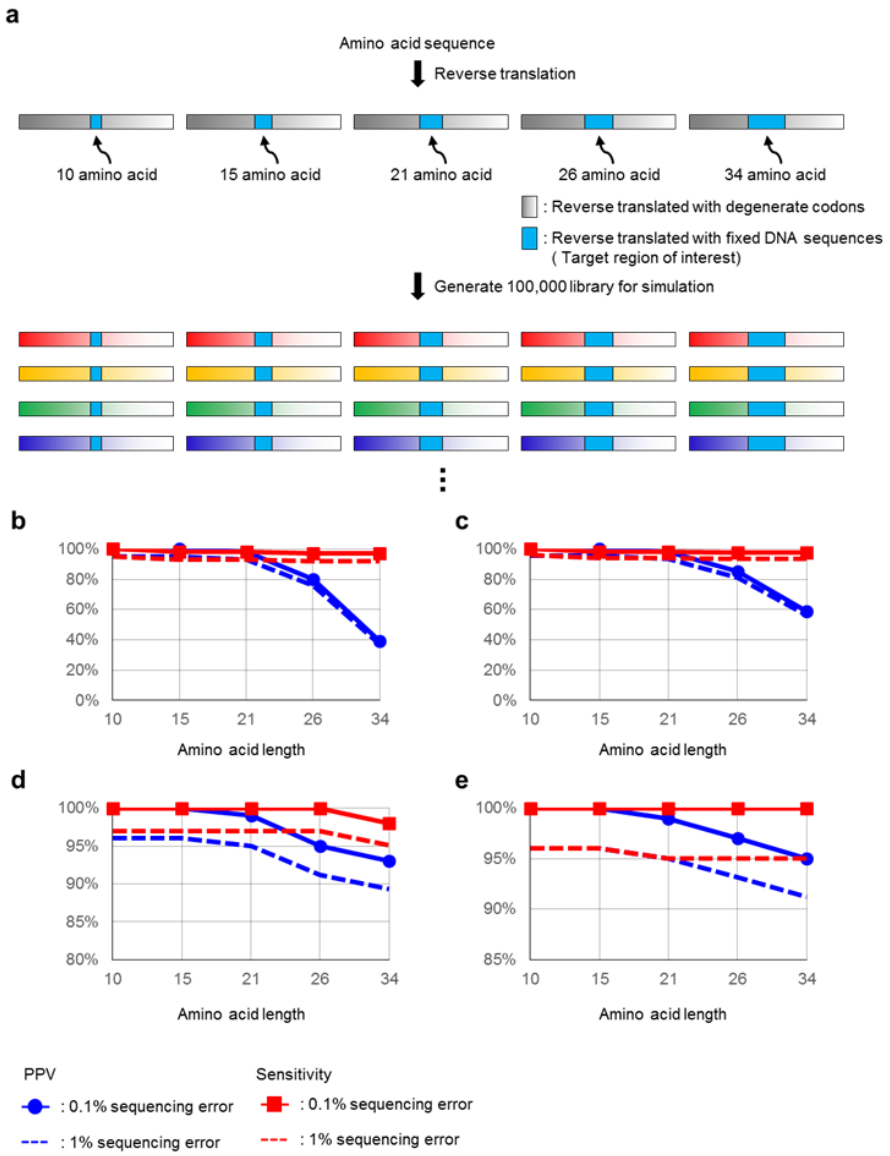

(a) We generated 100,000 normal PCR (0.1%, 1% sequencing error rate) libraries with a fixed DNA sequences region (not randomized to N, R, and Y, see figure above) within *tolC.* We performed the simulation (*k*-mer: 120) by varying the window size of the fixed region and compared two different barcoding strategies (using ‘N’ only or using ‘NRY’, assume 48x even coverage that showed high sensitivity in **Supplementary Fig. 9**). We observed decreased PPV value (an increase in false positives) for both (b, d) ‘N’ only and (c, e) ‘NRY’ strategies as the fixed length increased. We also generated random mutations to fixed region (blue) to have 1 mutation on average (range: 0-3). For both different barcoding scenarios (‘N’ for (d), ‘NRY’ for (e)), PPV increased to ~95%. Introducing 1% errors (substitution) on sequencing resulted in 2~3% decrease in PPV on average for above simulations.

Supplementary Figure 16. Simulation results of multiple-site (double) mutagenesis

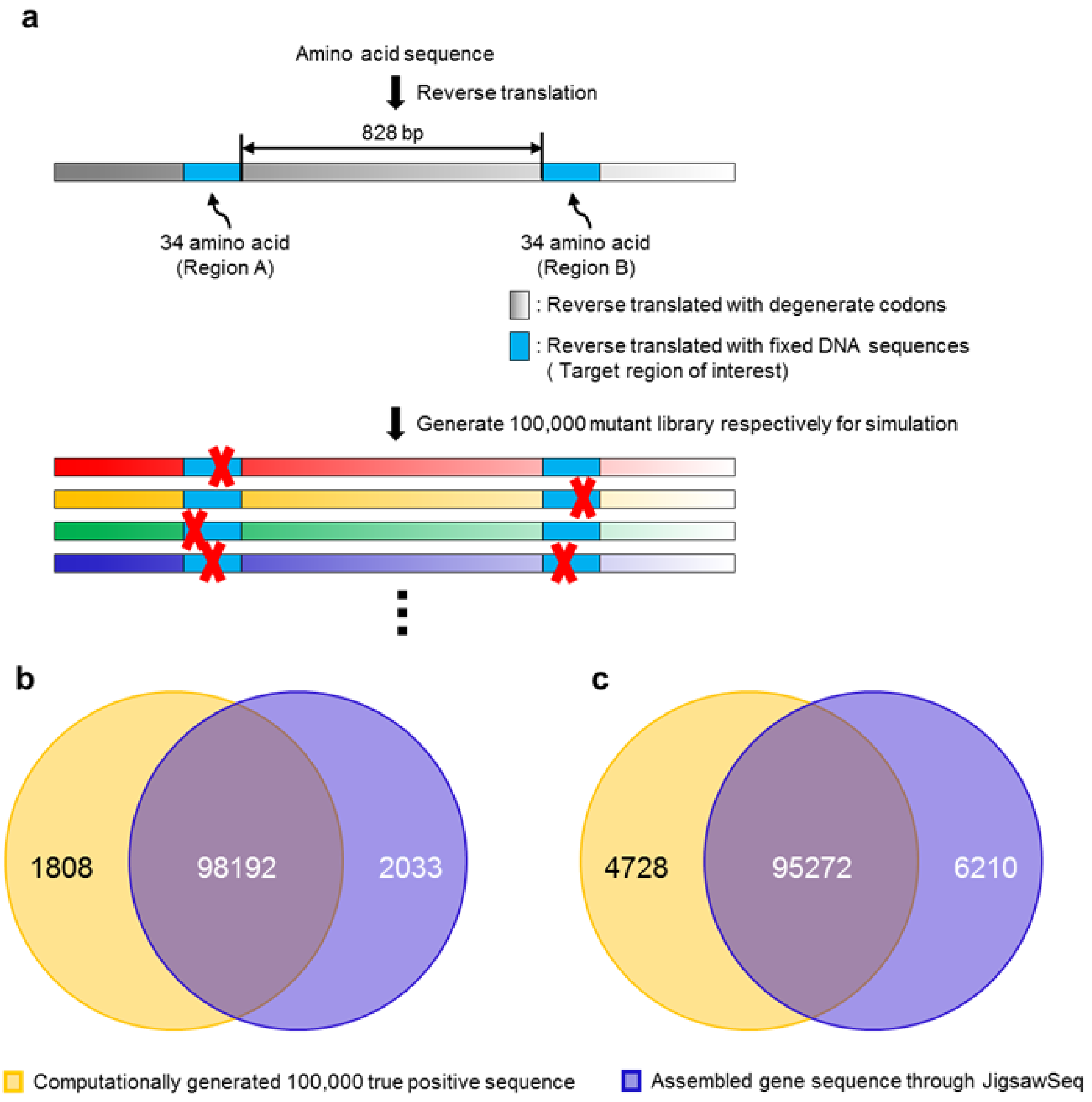

(a) We created mutated libraries of *tolC* including missense mutation as a singleton for two distant random regions. For the convenience of simulation, we limited the library size to 100,000. Venn diagram showed JigsawSeq's performance in the simulation when sequencing error rate is (b) 0.1% and (c) 1.0%. For both conditions, high concordance was observed between true positive contigs and called contigs from JigsawSeq. We could create the above library by either of the two methods discussed in **Supplementary Fig. 18**.

Supplementary Figure 17. Simulation results of single chain antibody (ScFv)

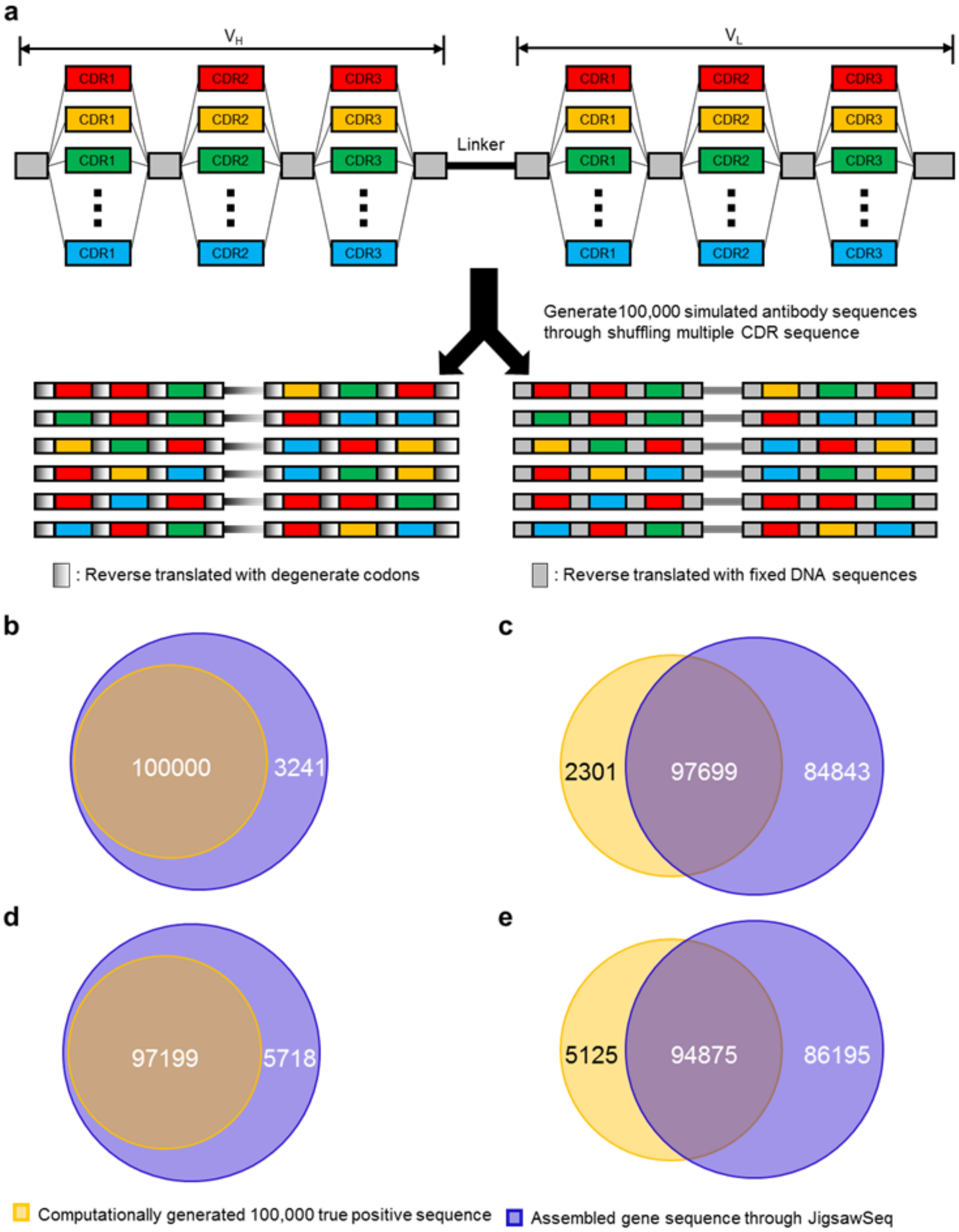

We focused on single-chain variable fragment, which is the simplest functional representation of an antibody molecule. To assess full-length antibody repertoires, we simulated 100,000 variable templates (sequencing error rate of 0.1%) by randomly shuffling multiple CDR sequences to mimic large genetic variation (a).

When comparing the library construction methods, the design (c, e) with fixed codons (a; gray region showing that the region is not randomized to ‘N’) showed poor performance in contrast with the randomized design (b, d). We observed slight decrease in sensitivity and increase in PPV when sequencing error rate is adjusted to 1%.

Supplementary Figure 18. JigsawSeq application to the library generated with different types of mutagenesis techniques

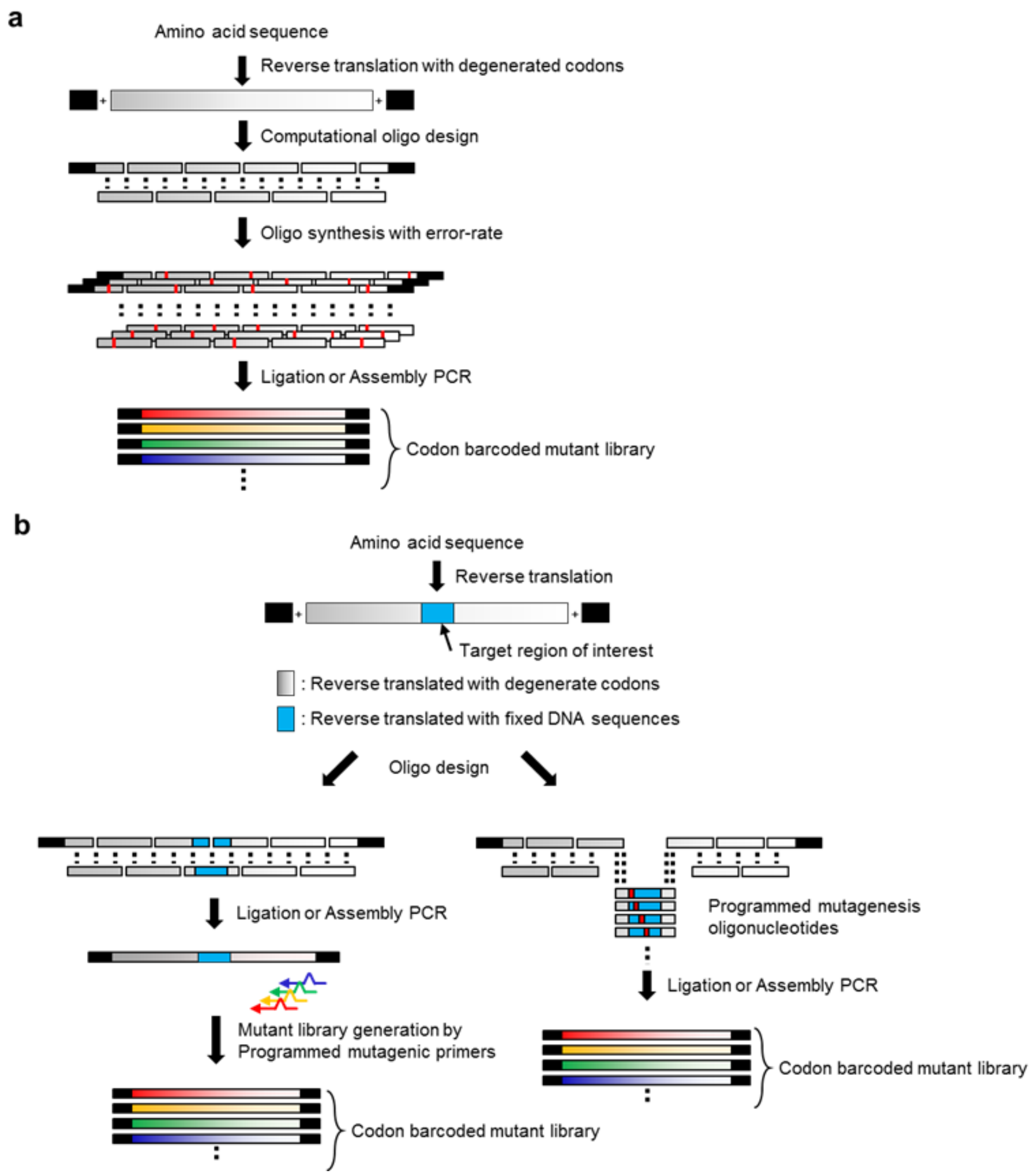

(a) Oligonucleotides could be synthesized with errors and then assembled to yield full-length gene products using ligation or assembly PCR. Each oligonucleotide sequence contains randomly distributed mutations and could be assembled to generate a codon-barcoded mutant library.

(b) Using a codon barcoded gene library, the practitioner could perform programmed mutagenesis on the target region of interest where DNA sequences are fixed (not randomized to 'N', 'R', and 'Y' sequences). The region excepting the fixed portion of sequences was randomized using degenerate bases. Mutagenic primers (left) or programmed mutant oligonucleotides (right) could generate mutations to specific fixed positions (blue).

Supplementary Figure 19. Results of selection of the initial *kanR* pool library

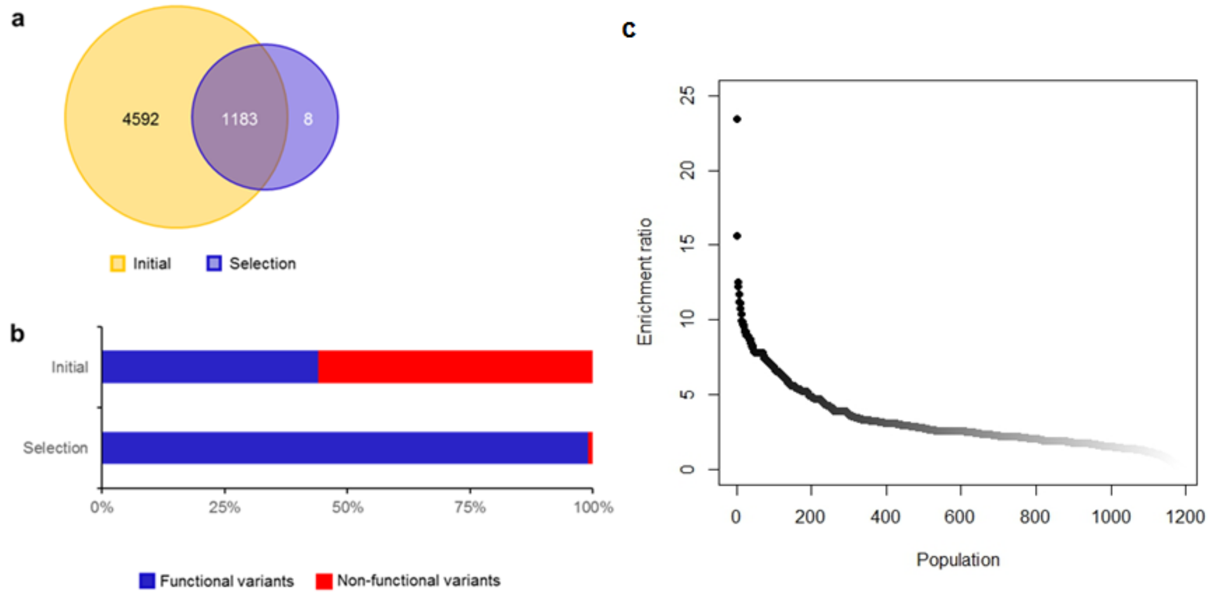

Comparing initial pool and selected pool (a), functional variants were highly enriched (b). The enrichment ratio is calculated as follows:

i: initial pool

s: selection pool

$$ER = \left[\frac{variant_{s,x}}{\sum_{s,x} variant_{s,x}} \middle/ \frac{variant_{i,x}}{\sum_{i,x} variant_{i,x}} \right]$$

($variant_{i,x}$) : depth of variant x in the initial pool

We plotted the enrichment ratio for intersecting population (1183) only (c). We reasoned that the eight remaining contigs in the selection pool would be due to the enriched population that was not sequenced in the initial pool since they contained rare alleles.

Supplementary Figure 20. Retrieval of *kanR* from the selection pool

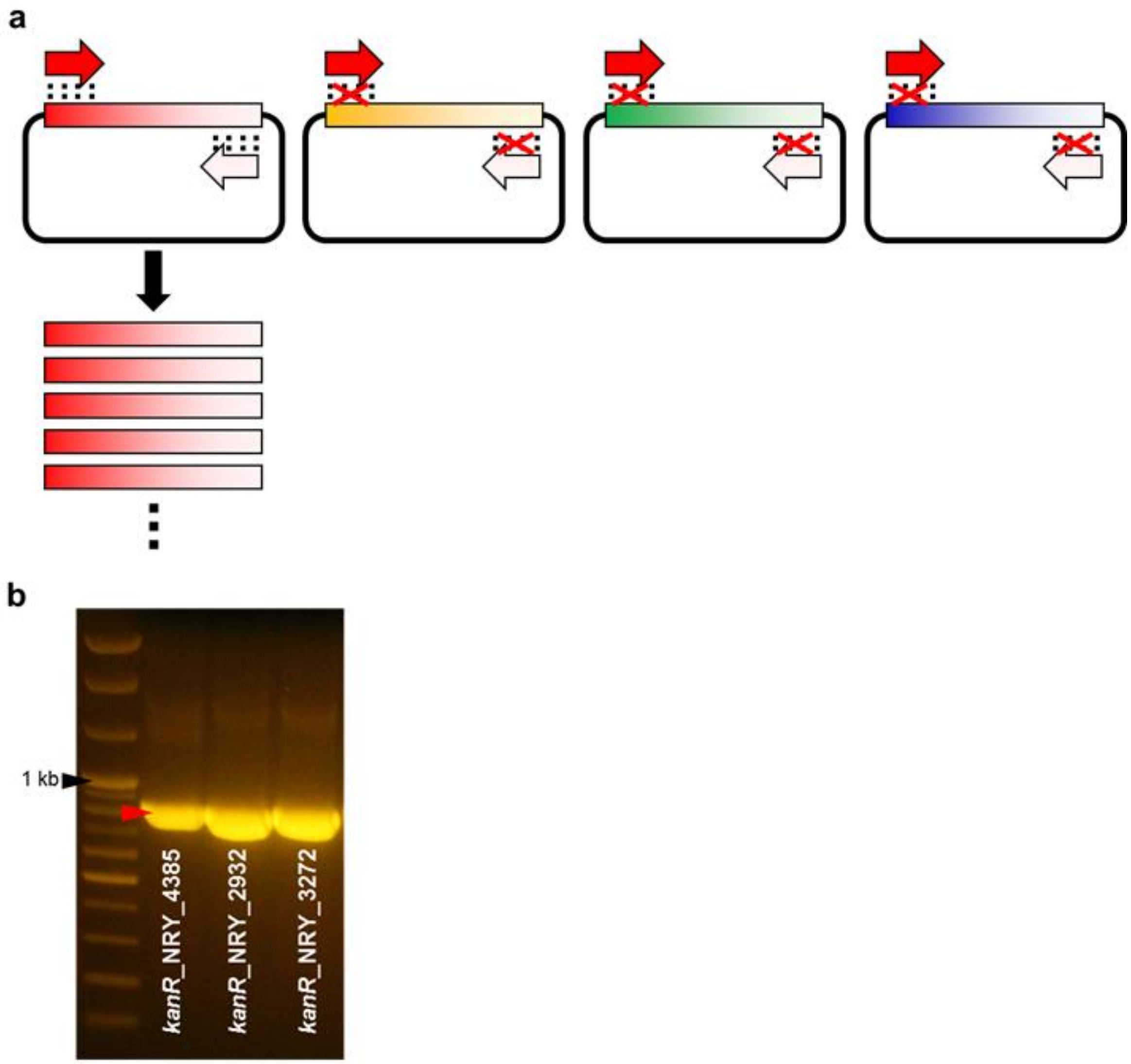

Specific primers containing molecular barcodes (‘N’, ‘R’ or ‘Y’) allow us to retrieve target of interest (a). For the validation, we have shown successful amplification (red arrow) of three variants out of three trials and these sequences were validated via Sanger sequencing (b).

Supplementary Fig. 21 Bioinformatics workflow of JigsawSeq

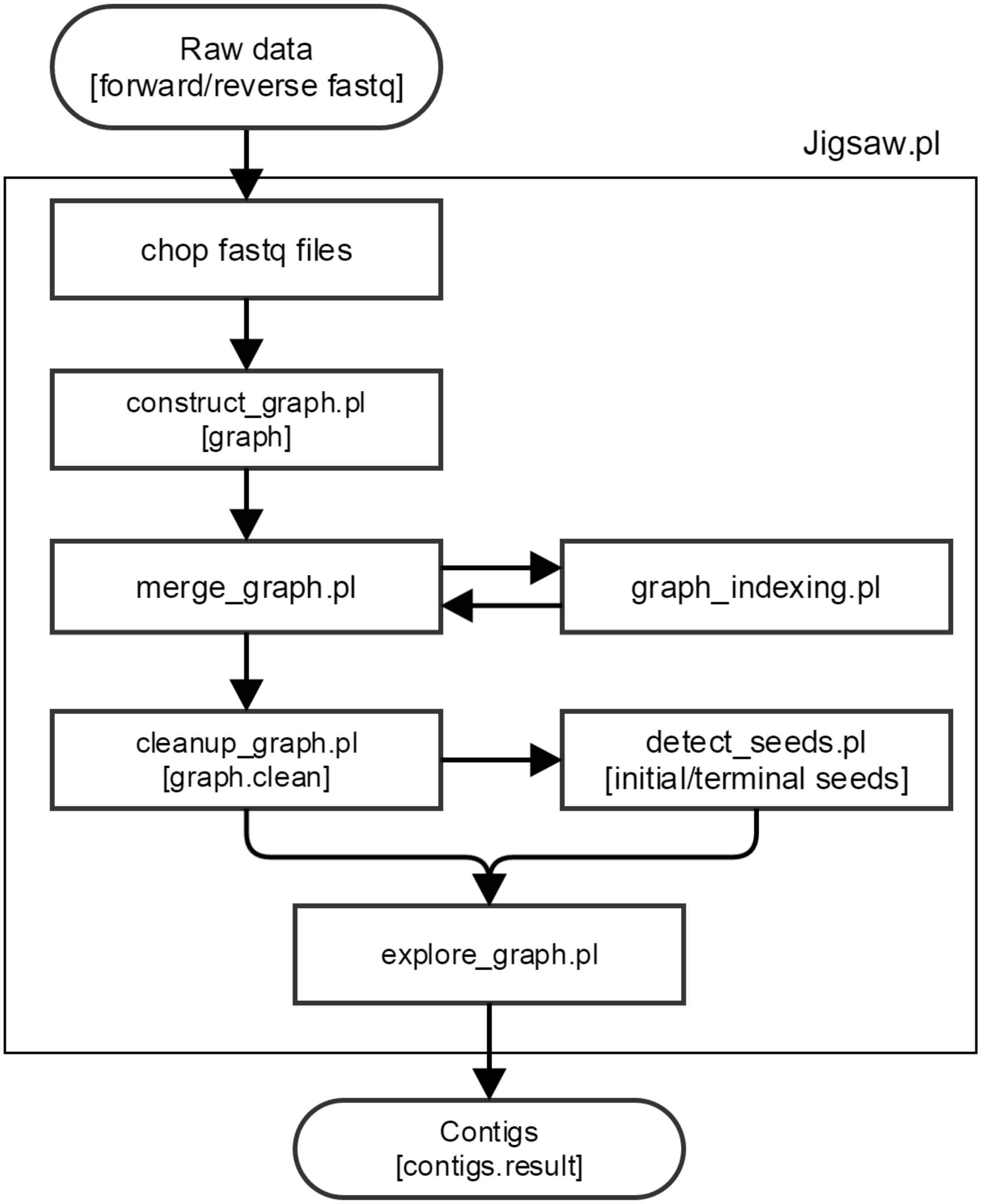

Supplementary Tables

Supplementary Table 1. Randomized genetic code used for constructing gene libraries.

a

Amino acid	codon	Amino acid	codon
Glycine(G)	GGN	Alanine(A)	GCN
Valine(V)	GTN	Leucine(L)	CTN
IsoLeucine(I)	ATY	Methionine(M)	ATG
Proline(P)	CCN	Serine(S)	TCN
Threonine(T)	CAN	Cysteine(C)	TGY
Phenylalanine(F)	TTY	Tyrosine(Y)	TAY
Tryptophan(W)	TGG	Aspartic acid(D)	GAY
Asparagine(N)	AAY	Glutamic acid(E)	GAR
Glutamine(Q)	CAR	Histidine(H)	CAY
Lysine(K)	AAR	Arginine(R)	CGN

b

Amino acid	codon	Amino acid	codon
Glycine(G)	GGN	Alanine(A)	GCN
Valine(V)	GTN	Leucine(L)	CTN
IsoLeucine(I)	ATT, ATC, ATA	Methionine(M)	ATG
Proline(P)	CCN	Serine(S)	TCN
Threonine(T)	CAN	Cysteine(C)	TGT, TGC
Phenylalanine(F)	TTT, TTC	Tyrosine(Y)	TAT, TAC
Tryptophan(W)	TGG	Aspartic acid(D)	GAT, GAC
Asparagine(N)	AAT, AAC	Glutamic acid(E)	GAA, GAG
Glutamine(Q)	CAA, CAG	Histidine(H)	CAT, CAC
Lysine(K)	AAA, AAG	Arginine(R)	CGN

Oligonucleotides were designed to show degeneracy by converting the third base of each codon to a mixed base.
(a) Method 1. Amino acid table for reverse translation with N, R, and Y random bases.
(b) Method 2. Amino acid table for reverse translation with N random base; I, F, N, Q, K, C, Y, D, E, and H were fixed to one of any possible codon changes by random selection.

Supplementary Table 2. Comparison of the assembly methods of different target genes

Target gene	Target size	Randomization Method (Randomized ratio)	Gene synthesis method	Backbone vector	Cloning method
Npu-intein*	411bp	N,R,Y(32.4%)	Assembly PCR	pBR322-du1	Enzyme digestion & Ligation
kanR	816bp	N,R,Y(31.5%)	LCR	pBR322-du1	Gibson assembly
mcardinal	735bp	N(16.3%)	LCR, Assembly PCR	pEGFP-C1	Gibson assembly
*tolC**	1,482bp	N(19.0%)	Assembly PCR	pBR322-du1	Gibson assembly

(*) represents a library constructed with error-prone PCR. We have listed all properties for various gene libraries; in the third column, the randomized ratio is calculated by dividing total N, R, and Y bases by the total length of the gene. For *mcardinal*, we used both LCR and assembly PCR methods for gene variant library construction.

Supplementary Table 3. Variant distribution in Sub Sanger pools

Pool	Gene size (bp)	Functional (%)	Non-functional (%)
Npu-intein_Sub	411	58	42
*mcardinal*_Sub (AssemblyPCR)	735	30	70
*mcardinal*_Sub (LCR)	735	41	59
*kanR*_Sub	816	44	56
*tolC*_Sub	1482	18	82

Generally, the frequency of functional variants (in-frame contigs without premature stop codons) decreases with assembled gene size. Non-functional variants are contigs with a length of 3n+1, 3n+2, or 3n (In-frame) length with a nonsense mutation (substitution) or indel.

Supplementary Table 4. Estimating computational requirements for real data

Pool	Data (GB)	Peak memory usage (G)	Running time (h)
Npu-intein_Sub	0.39 (1.3)	2.4	0.19
*mcardinal*_Sub (Assembly PCR)	0.78 (2.6)	3.6	0.29
*mcardinal*_Sub (LCR)	0.57 (1.9)	1.6	0.26
*kanR*_Sub	0.54 (1.8)	2.6	0.25
*tolC*_Sub	0.40 (2)	2.4	0.16
*kanR*_initial	13.8 (65)	20.2	7.1
*tolC*_initial	19.6 (65)	15.3	12.1

Values in the parentheses () refers to the original raw data. The rest of the values are calculated based on removal of the backbone data. Running time depends on several factors including data size and graph complexity. Peak memory size relies on the number of nodes or *k*-mer multiplicity, which represents the complexity of the path.

Supplementary Table 5. Performance optimization of sub pool libraries using JigsawSeq

Pool	With alignment		Before alignment with adjusted parameter	
	Sensitivity (%)	PPV (%)	Sensitivity (%)	PPV (%)
Npu-intein_Sub	96.2	89.4	96.2	89.4
*mcardinal*_Sub (Assembly PCR)	88.5	94.5	94.8	83.1
*mcardinal*_Sub (LCR)	91.5	92.9	90.1	91.4
*kanR*_Sub	88	97.6	92.3	95.5
*tolC*_Sub	87.3	95.4	91.5	95.5

Overall, for sub pool gene variant libraries, the coefficient of variation (CV) filter with the BWA alignment step allows for lower sensitivity and higher PPV. As the aligning step is time-consuming, we modified the algorithm to make the final filter step optional. If the user requires high PPV, the filtering step can be enabled by turning on the -a (--realign) option. The adjusted parameters were --cut_edge 50, --cut_seed 200. A detailed description of the parameters is provided on the website (https://github.com/jy2/JigsawSeq).

Supplementary Table 6. Primers used for the retrieval of *kanR* sequences

	For primer	Rev primer
kanR_NRY_4385	ATGTCTCATATCCAACGCGAGACC	TTAAAAGAACTCATCAAGCATT
kanR_NRY_2932	ATGTCGCATATTCAACGTGAAACT	TTAGAAAAACTCATCTAGCATC
kanR_NRY_3272	ATGTCTCATATCCAACGGGAGACC	TTAAAAGAATTCGTCTAGCATC
